# Age-specific nasal epithelial responses to SARS-CoV-2 infection

**DOI:** 10.1038/s41564-024-01658-1

**Published:** 2024-04-15

**Authors:** Maximillian N. J. Woodall, Ana-Maria Cujba, Kaylee B. Worlock, Katie-Marie Case, Tereza Masonou, Masahiro Yoshida, Krzysztof Polanski, Ni Huang, Rik G. H. Lindeboom, Lira Mamanova, Liam Bolt, Laura Richardson, Batuhan Cakir, Samuel Ellis, Machaela Palor, Thomas Burgoyne, Andreia Pinto, Dale Moulding, Timothy D. McHugh, Aarash Saleh, Eliz Kilich, Puja Mehta, Chris O’Callaghan, Jie Zhou, Wendy Barclay, Paolo De Coppi, Colin R. Butler, Mario Cortina-Borja, Heloise Vinette, Sunando Roy, Judith Breuer, Rachel C. Chambers, Wendy E. Heywood, Kevin Mills, Robert E. Hynds, Sarah A. Teichmann, Kerstin B. Meyer, Marko Z. Nikolić, Claire M. Smith

**Affiliations:** 1 Great Ormond Street UCL Institute of Child Health, London, UK; 2 Wellcome Sanger Institute, Cambridge, UK; 3 UCL Respiratory, Division of Medicine, University College London, London, UK; 4 UCL Institute of Ophthalmology, University College London, London, UK; 5 Royal Brompton Hospital, Guy’s and St Thomas’ NHS Foundation Trust, London, UK; 6 UCL Centre for Clinical Microbiology, Division of Infection and Immunity, University College London, London, UK; 7 Royal Free Hospital NHS Foundation Trust, London, UK; 8 University College London Hospitals NHS Foundation Trust, London, UK; 9 Department of Infectious Disease, Imperial College London, London, UK; 10 Great Ormond Street Hospital NHS Foundation Trust, London, UK; 11 Epithelial Cell Biology in ENT Research (EpiCENTR) Group, Developmental Biology and Cancer Department, Great Ormond Street UCL Institute of Child Health, University College London, London, UK; 12 UCL Cancer Institute, University College London, London, UK; 13 Theory of Condensed Matter, Cavendish Laboratory/Dept Physics, University of Cambridge, Cambridge, UK

**Keywords:** SARS-CoV-2, Molecular biology, Mechanisms of disease

## Abstract

Children infected with SARS-CoV-2 rarely progress to respiratory failure. However, the risk of mortality in infected people over 85 years of age remains high. Here we investigate differences in the cellular landscape and function of paediatric (<12 years), adult (30–50 years) and older adult (>70 years) ex vivo cultured nasal epithelial cells in response to infection with SARS-CoV-2. We show that cell tropism of SARS-CoV-2, and expression of ACE2 and TMPRSS2 in nasal epithelial cell subtypes, differ between age groups. While ciliated cells are viral replication centres across all age groups, a distinct goblet inflammatory subtype emerges in infected paediatric cultures and shows high expression of interferon-stimulated genes and incomplete viral replication. In contrast, older adult cultures infected with SARS-CoV-2 show a proportional increase in basaloid-like cells, which facilitate viral spread and are associated with altered epithelial repair pathways. We confirm age-specific induction of these cell types by integrating data from in vivo COVID-19 studies and validate that our in vitro model recapitulates early epithelial responses to SARS-CoV-2 infection.

## Abstract (web summary)

Age-specific differences upon SARS-CoV-2 infection are marked by emergence of goblet 2 inflammatory cells expressing antiviral interferon stimulating genes in paediatric nasal cultures, and basaloid-like cells with increased viral spread in cultures from older adults.

## Main

Despite effective vaccines, age remains the single greatest risk factor for COVID-19 mortality. Children infected with severe acute respiratory syndrome coronavirus 2 (SARS-CoV-2) rarely develop severe disease^1^, while the mortality in infected people over 85 years is currently as high as 1 in 10 (ref. ^2^). Nasal epithelial cells (NECs) are the primary target of SARS-CoV-2 (refs. ^3,4^), and understanding their viral response is crucial as infection of upper airway cells can progress distally^5,6^, leading to diffuse alveolar injury with respiratory failure and long-term complications including lung fibrosis^7^.

Initially, it was thought that higher viral entry factor expression of angiotensin-converting enzyme 2 (ACE2) and transmembrane serine protease 2 (TMPRSS2) in adults could explain increased severity, but such differences between children and adults remain uncertain^8,9^. Children may alternatively be protected by a pre-activated antiviral state in the upper airways^9,10^, but this does not fully explain the increased risk with increasing age. In addition, most in vivo studies so far were unable to identify early cellular responses, since in almost all cases the exact time of infection was unknown, symptom onset was variable and research sampling usually occurred only a few days after testing positive for SARS-CoV-2 (ref. ^9^).

Here we investigated the effects of early SARS-CoV-2 infection on human NECs from healthy children (0–11 years), adults (30–50 years) and older adults (>70 years). NEC were cultured at an air-liquid interface (ALI) and either subjected to mock infection or infected with SARS-CoV-2 for up to 3 days. This setup was used to examine epithelial-intrinsic differences in function, viral replication, gene and protein expression. We reveal age-specific epithelial responses, independent of immune cells, with a strong interferon (IFN) response in infected paediatric goblet inflammatory cells, and the appearance of older adult basaloid-like cells that sustain viral replication and are associated with fibrotic signalling pathways.

## Results

### Differences in the cellular landscape of NECs with age

**Fig. 1:**
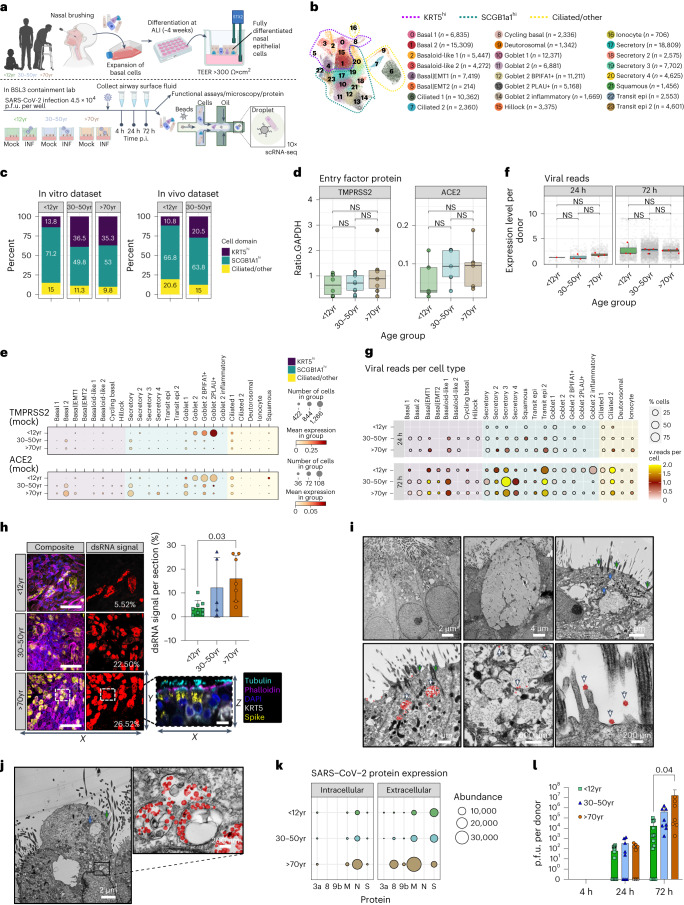
Characterization of SARS-CoV-2-infected NEC cultures from different age groups. **a**, Schematic of method and model used to study SARS-CoV-2 infection of paediatric (P, <12 years), adult (A, 30–50 years) and older adult (O, >70 years) nasal epithelial cells. **b**, UMAP visualization of annotated airway epithelial cells. Cell numbers per cell type are shown in parentheses. Dotted lines indicate the three principal cell domains these fall within: KRT5 high (KRT5^hi^), SCGB1A high (SCGB1A^hi^) and ciliated/other. UMAP shows the entire single-cell sequencing (scRNA-seq) dataset, including SARS-CoV-2 and mock-infected NEC cultures across all three timepoints and ages (*n* = P3, A4, O4). **c**, Percentage of annotated airway epithelial cells with respect to age in baseline (non-infected) NEC cultures and following label transfer to an in vivo dataset of nasal brushings from age-matched donors from ref. ^9^ (data shown as a percentage cells in the three principal cell domains found in each age group). **d**, SARS-CoV-2 entry factor protein expression per culture type determined by Western blot. Comparisons of ACE2 and TMPRSS2 protein levels normalized to GAPDH were made using the Wilcoxon test. Individual values plotted for each participant, indicated by dots (*n* = P9, A7, O8). **e**, SARS-CoV-2 entry factor gene expression by scRNA-seq. SARS-CoV-2 entry factor gene expression per cell type calculated on the basis of absolute cell numbers, with the average expression of *ACE2* and *TMPRSS2* indicated by colour. Dot size corresponds to the number of cells expressing *ACE2* and *TMPRSS2* in respective age groups in the mock condition. **f**, SARS-CoV-2 RNA viral reads (grey dots, per cell; red dots, per donor) as determined by viral transcript counts (encoding for the full viral genome) per nucleotide per 500 cells (grey dots) or nucleotide per 500 cells per donor (red dots) within each age group. Pairwise comparisons between donors’ age groups were performed using two-sided Wilcoxon rank-sum tests; NS, not significant. **g**, SARS-CoV-2 viral reads were detected within the scRNA-seq dataset (Infected condition only) at 24 (top) and 72 h (bottom) post infection, shown by cell type and age groups, with dot size and colour indicative of the percentage of cells with detectable viral reads and average reads per cell, respectively. **h**, Representative maximum intensity *z*-projections of confocal images (left) of NEC cultures immunolabelled against cilia (cyan, tubulin), dsRNA (yellow) and basal cells (KRT5, white) with DAPI (blue) and phalloidin (magenta) to indicate the nucleus and actin filaments, respectively. Scale bar, 50 μm. Representation of dsRNA signal alone for each section is indicated in red adjacent to respective maximal projections, with the value of spread given on each panel. Summarized on the bar graph to the right (mean ± s.d.), subjected to one-way analysis of variance (ANOVA) with Tukey’s multiple comparisons test. Individual values are shown for each donor (*n* = P8, A5, O6). A representative orthogonal section is given (bottom right) to indicate location of dsRNA within infected NECs. **i**,**j**, Transmission electron micrographs of epithelial cell types infected with SARS-CoV-2, with selected areas of interest shown at a higher magnitude for each; **i**, ciliated cells (left), goblet cell (middle), transit (right) and **j**, ciliated 2 cell types. Panels show components of interest within each cell type, denoted by arrows: white arrows, SARS-CoV-2; green arrows, cilia; blue arrows, secretory mucin granules; viral particles false-coloured with red to aid visualization. **k**, SARS-CoV-2 protein abundance in apical fluid (extracellular) and cell lysates (intracellular) from SARS-CoV-2-infected NECs for 72 h p.i. as determined by mass spectrometry. Data are shown as mean abundance of protein (dot size) and mean fold change (FC) in protein abundance per donor from mock-infected NECs (colour, age group) (*n* = P5, A5, O5). **l**, Infectious viral titres in combined cell lysate and apical fluid of SARS-CoV-2 nasal epithelial cells from paediatric, adult and older adult donors as determined by plaque assays (mean ± s.d.). Two-way ANOVA with Tukey’s multiple comparisons test. Individual values are shown for each donor (*n* = P13, A8, O8). Lines in box and whisker plots (**d**,**f**) indicate median, interquartile range (IQR) and minimum to maximum of the distribution. Source data

**Extended Data Fig. 1:**
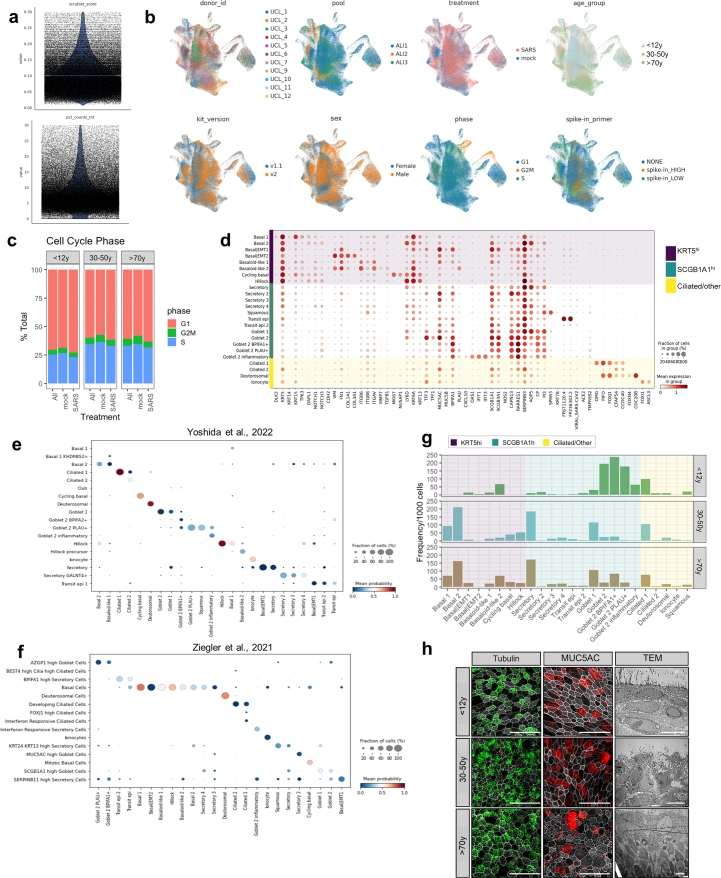
Paediatric nasal epithelial cells have less basal cell subtypes compared to adult and older adult nasal epithelial cells but display comparable differentiation markers and SARS-CoV-2 entry factor expression. **(a)** Violin plot visualization of threshold for percentage of mitochondrial reads in scRNAseq dataset. **(b)** Uniform manifold approximation and projection (UMAP) visualizations showing good integration of donor IDs, donor pool, treatment, age group, time after infection, 10X Chromium kit version, sex, cell cycle phase and introduced spike-in primer after batch correction (see Methods for more details). **(c)** Boxplot indicating comparison of cell cycle phase states (G1, G2M, S) amongst the three age groups in mock/infected/combined (All) conditions. **(d)** Dot plot visualization showing marker genes for annotated airway epithelial cell types, with fraction of expressing cells and average expression within each cell type indicated by dot size and colour, respectively. Broad cell domains are colour coded; KRT5^hi^ (purple), SCGB1A1^hi^ (green) and ciliated/other (yellow). Logistic regression based label transfer using Celltypist for the data sets in **(e)** Yoshida et al.^9^ and **(f)** Ziegler et al.^12^, with fraction of matched cells and average probability score indicated by dot size and colour, respectively **(g)** Numbers of annotated airway epithelial cells in respect to age (data shown in ratio of cell numbers/per 1000 cells in age dataset). **(h)** Representative maximum intensity z-projections of confocal images (left and central) and transmission electron micrographs (right panels) of NEC cultures differentiated at an air–liquid interface and immunolabeled against cilia (tubulin, green) and mucin (MUC5AC, red), with DAPI (blue) and phalloidin (grey) to indicate the nucleus and actin filaments, respectively. Scale bar 10-μm applies to all images in the row. Source data

We first investigated the cellular composition of NECs at different ages using single-cell RNA sequencing (scRNA-seq; Fig. 1a). We analysed a dataset of 139,598 cells and identified 24 distinct epithelial cell types or states (Fig. 1b and Extended Data Fig. 1a–c). These included basal (KRT5^hi^), secretory (SCGB1A1^hi^, MUC5AC+) and ciliated (CCDC40+) cells (markers in Extended Data Fig. 1d). Basal cells encompassed various subpopulations, such as basal, cycling basal, hillock, basal|EMT (associated with epithelial–mesenchymal transition (EMT)) and basaloid-like cells enriched in fibrotic lungs^11^. The second domain includes secretory, goblet and squamous cells, each expressing different secretory proteins and genes related to mucosal defence. The third domain comprised ciliated cells, which were further divided into two clusters on the basis of gene expression patterns associated with cilium organization. Comparison to published nasal COVID-19 datasets^9,12^ confirmed the accuracy of our cell annotations including ionocytes and hillock cells (Extended Data Fig. 1e,f).

**Extended Data Fig. 2:**
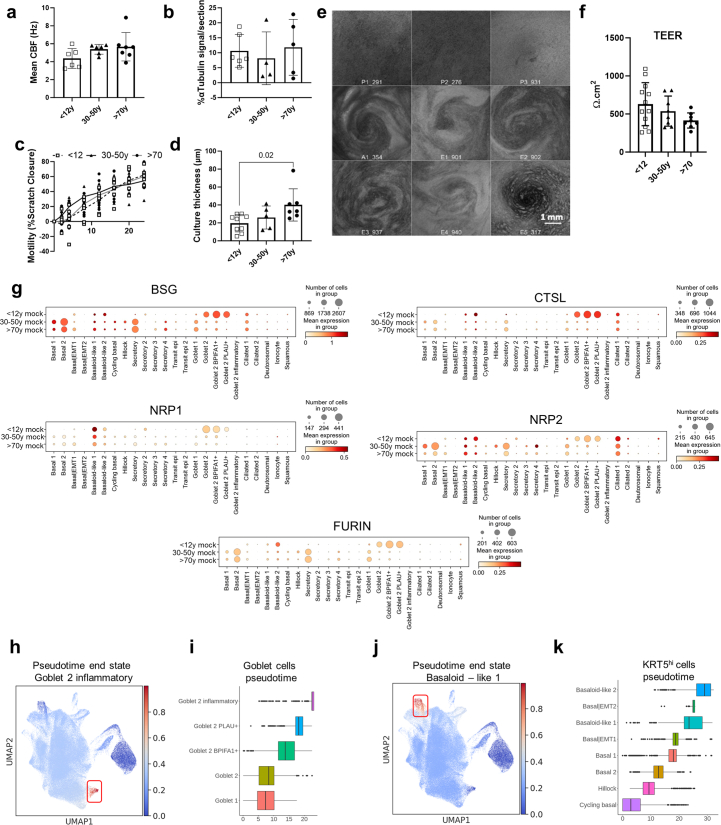
Physiological differences in paediatric, adult and older adult nasal epithelial NEC cultures. Physiological comparison of paediatric (P), adult (A) and older adult (O) NEC cultures as measure by; **(a)** ciliary beat frequency (CBF)(Hz) (n = P6, A6, O7); **(b)** the percentage of +ve ɑ-tubulin staining coverage per 225 μm2 section (n = P6, A4, O5); **(c)** motility measured by percentage scratch closure over time (n = P8, A3, O8) and **(d)** culture thickness (n = P9, A5, O7). For (**a-d**) data was plotted as mean ± SD, subject to one-way ANOVA with Tukey’s multiple comparison test **(e)** Representative light microscope images of whole well scans of NEC cultures depicting the characteristic differences in culture morphology with age (P=Paed, A=Adult, E=Older adult). **(f)** Comparison of epithelial integrity via trans-epithelial electrical resistance (TEER) (Ω.cm^2^). Mean ± SD (n = P12, A8, O8) plotted across the age groups using one-way ANOVA with Tukey’s correction. **(g)** Alternate SARS-CoV-2 entry factor gene expression per cell type calculated based upon absolute cell numbers with the average expression of *BSG, CTSL, NRP1, NRP2* and *FURIN* indicated by colour. Dot size corresponds to the total number of cells expressing alternate viral entry genes in respective age groups in the mock condition. (**h**) Palantir inferred probabilities of cycling basal cells differentiating into basaloid-like 2 or goblet 2 inflammatory cells. **(i)** Boxplot showing the distribution of pseudotime within each cluster among goblet cell subtypes. **(j)** Palantir inferred probabilities of cycling basal cells differentiating into basaloid-like 2 cells. **(k)** Boxplot showing the distribution of pseudotime within each cluster among KRT5^hi^ cell subtypes. Lines in box and whisker plots **i, j** indicates median, interquartile range, and minimum to maximum of the distribution, with individual values for each cell shown. Source data

Interestingly, we observed age-related differences in cell-type proportions in healthy control cultures, with a higher abundance of basal/progenitor subtypes in adult versus paediatric cultures (Fig. 1c and Extended Data Fig. 1g), mirroring an in vivo nasal epithelial dataset^9^ (Extended Data Fig. 1e,f). All age groups exhibited similar apical differentiation, mucus and tubulin expression (Extended Data Fig. 1h), and ciliary activity (Extended Data Fig. 2a,b). There was no substantial difference in ciliary beat frequency or cellular motility with age (Extended Data Fig. 2a,c). However, NEC cultures from older adult donors were thicker (mean ± s.d. 40 ± 18 µm) than paediatric cultures (20 ± 10 µm; *P* = 0.02) (Extended Data Fig. 2d) with a distinct spiral morphology typical of NEC cultures (Extended Data Fig. 2e), though this had no effect on the integrity of the epithelial barrier (Extended Data Fig. 2f).

The most notable difference in paediatric cultures was an increase of goblet cell types, particularly the goblet 2 cells (Extended Data Fig. 1g). This shift in cell state from secretory (higher in *KRT5*) to goblet (higher in *BPIFA1)* cells was not observed in adult and older adult cultures. Importantly, while the total protein levels of SARS-CoV-2 entry factors^13^ did not vary with age (Fig. 1d), paediatric cultures showed higher mRNA expression of *TMPRSS2* and *ACE2* in goblet cells (Fig. 1e). In adult and older adult cultures, these markers were predominantly expressed in secretory and basal 2 cell types (Fig. 1e), suggesting a shift in susceptibility to viral infection from goblet to secretory cell types with age. Other viral entry factors, *BSG*, *CTSL*, *NRP1*, *NRP2* and *FURIN* showed the same trend as *ACE2* and *TMPRSS2* (Extended Data Fig. 2g).

### Increased virus production in infected older adult NECs

**Extended Data Fig. 3:**
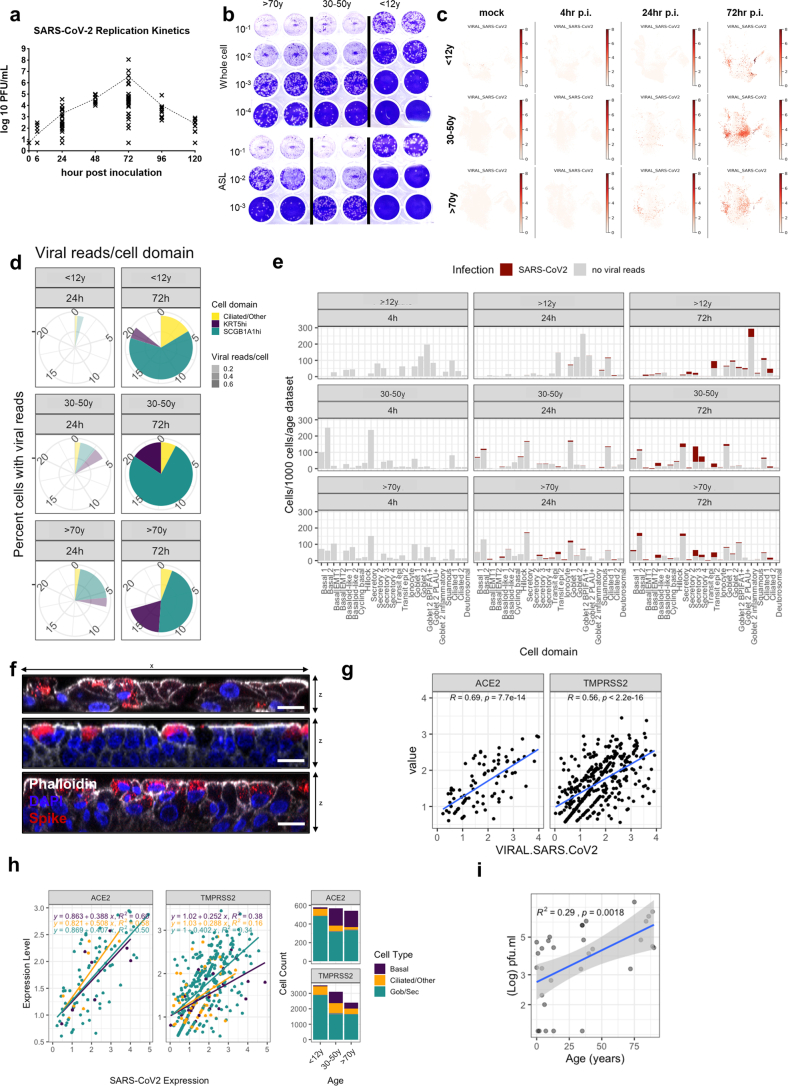
Elderly cells replicate more infectious viruses with a greater distribution of viral reads across epithelial subtypes. **(a)** Mean viral replication shown as pfu/ml per donor over a 5-day period (n = 35: P15, A10, O10). **(b)** Exemplar image of plaque assay used to determine infectious viral load. **(c)** UMAP of airway cells with detected SARS-CoV2 mRNA in each cell type in cultures mock and SARS-CoV-2 infected for all time points and ages combined (≥1 viral UMI per donor following filtering out ambient RNA). **(d)** Pie charts showing the fractions of annotated airway epithelial cells containing viral reads at 4 h, 24 h and 72 h p.i. in respect to age. **(e)** Numbers of annotated airway epithelial cells containing viral reads at 24 h and 72 h post infection (red) in respect to age with total number of cells in each subset (grey) (data shown as cell numbers/per 1000 cells in age dataset). **(f)** Representative orthogonal views of z-stacks from fixed paediatric (top), adult (middle) and older adult (bottom) NECs at 72 h p.i. with SARS-CoV-2. Sections were immunolabeled against F-actin (phalloidin, grey) DAPI (blue) and SARS-CoV-2 S protein (red). The scale bar represents 10 μm. **(g)** Linear regression analysis of viral reads vs ACE2 and TMPRSS2 expression per cell at 72 h post infection. Data represents all age groups combined **(h)** Linear regression analysis of viral reads vs ACE2 and TMPRSS2 expression per cell and grouped by cell domains **(i)** Linear regression of infectious viral titres in combined cell lysate and apical fluid of SARS-CoV-2 nasal epithelial cells from donors of different ages as determined by plaque assays (n = 29). Subject to Pearson correlation with SE shown for line error. Source data

To determine differences in viral replication between age groups, NEC cultures were infected with an early-lineage SARS-CoV-2 isolate (hCoV-19/England/2/2020; 4 × 10^4^ plaque forming units (p.f.u.) per well (approximate multiplicity of infection (MOI) of 0.01 p.f.u. per cell)). Over a 5-day infection period, SARS-CoV-2 replication increased and then peaked at 72 h post infection (p.i.) (Extended Data Fig. 3a,b); therefore, all subsequent investigations were completed before this timepoint. The total number of viral reads increased with time but did not differ between age groups (Fig. 1f), with fewer cell types infected (showing >0 viral reads) in paediatric (3/24 cell types) versus adult and older adult cultures (7/24 and 11/24 cell types, respectively) at 24 h p.i. (Fig. 1g and Extended Data Fig. 3c–e) and a wider range at 72 h p.i. in all age groups (Fig. 1g). We also measured total viral spread (measured as %dsRNA+ signal coverage) by immunofluorescent analysis at 72 h p.i., which was greater in older adult (mean ± s.d. 16.1% ± 9.5) than in paediatric cultures (3.8% ± 3.1) (Fig. 1h and Supplementary Fig. 1). Overall, ciliated 2 and transit epi 2 cells had the highest proportion of viral reads (Fig. 1g). Strikingly, goblet cell types appeared more infected in paediatric cultures, while adult and older adult cultures showed highest viral reads in secretory cell types (Fig. 1g and Extended Data Fig. 3c–e). Cells expressing the highest viral reads displayed high ACE2 (*R*^2^ = 0.71) and TMPRSS2 (*R*^2^ = 0.57) expression (Extended Data Fig. 3f,g). Transmission electron microscopy (TEM) demonstrated the presence of viral particles (red) in cells possessing both mucin-containing secretory granules and cilia (Fig. 1i,j, and Supplementary Figs. 2 and 3).

Key differences across the age groups were greater apical localization of the SARS-CoV-2 spike protein (Extended Data Fig. 3f), greater abundance of intracellular and apical secreted SARS-CoV-2 proteins (Fig. 1k) and higher levels of infectious particles in older adult than in with paediatric cultures, with a significant (*P* = 0.04) >800-fold higher titre in older adult (mean ± s.d. 1.64 × 10^7^ ± 3.94 × 10^7^ p.f.u. per well; *n* = 8) than in paediatric cultures (1.71 × 10^4^ ± 3.20 × 10^4^ p.f.u. per well; *n* = 13) at 72 h p.i. (Fig. 1l and Extended Data Fig. 3i). These findings support the conclusion that SARS-CoV-2-infected older adult NECs translate more viral protein and generate more replication-competent viruses compared with paediatric cells.

### SARS-CoV-2 infection induces age-specific effects

We next profiled the phenotypic effects of infection on epithelial cells, using live cell microscopy, immunofluorescence staining, proteomics and gene expression analysis, and compared these across the age groups.

**Fig. 2:**
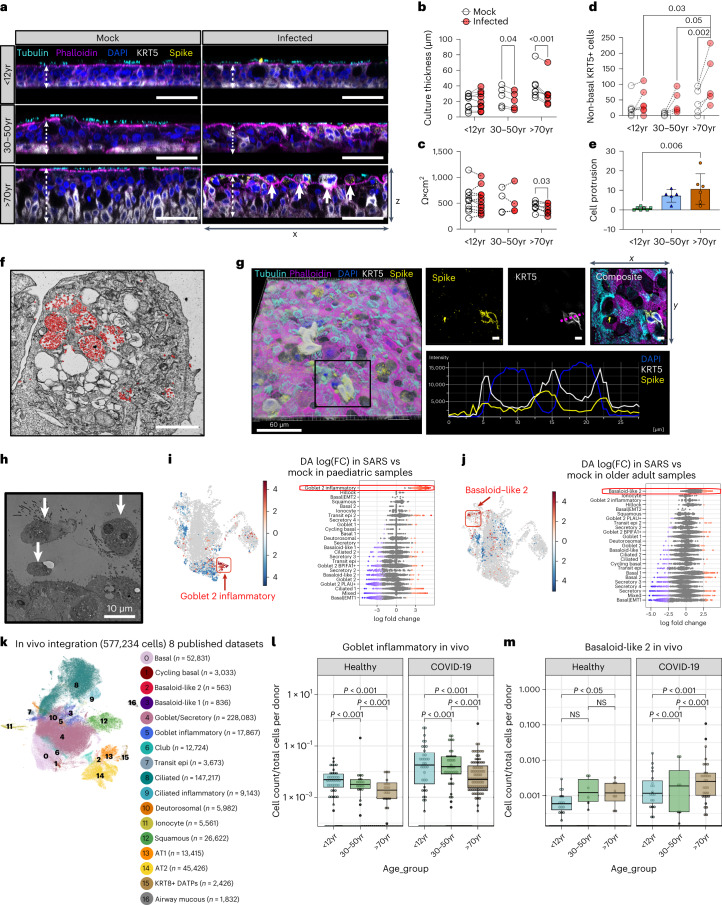
Cytopathology and cellular changes following SARS-CoV-2 infection of NECs. **a**, Representative orthogonal views of the *z*-stacks showing the thickness (white dashed arrow) and morphology of fixed paediatric, adult and older adult mock- or SARS-CoV-2-infected NECs at 72 h p.i. Sections were immunolabelled against cilia (cyan, tubulin), F-actin (magenta, phalloidin), DAPI (blue), SARS-CoV-2 S protein (yellow) and cytokeratin 5 (white, KRT5+). Solid white arrows indicate cells protruding from the apical surface (as quantified further in **e**). Scale bar, 50 μm. **b**, Epithelial thickness was further measured and quantified, and subjected to a two-way ANOVA with Sidak’s multiple comparison test (*n* = P9, A5, O7). **c**, Epithelial integrity, as measured by trans-epithelial electrical resistance (TEER) (Ω × cm^2^) from 72 h p.i. mock- or SARS-CoV-2-infected NECs (*n* = P11, A4, O7), subjected to multiple paired *t*-tests. **d**, Quantification of non-basal KRT5+ cells (for example, KRT5+ cells above and not touching the basal membrane) as a measure of basal cell mobilization, with age and infection (mock vs infection). Calculated using a cross-section of fixed NECs at 72 h p.i. (*n* = P7, A5, O5), subjected to two-way ANOVA with Tukey’s multiple comparisons test. See Extended Data Fig. 4c,d for more details for analysis. **e**, Cell protrusion analysis, calculated by counting the number of nuclei (blue, indicated by white solid arrows in **a**) above apical epithelial membrane (magenta) per section per donor. Data shown as mean ± s.d. (*n* = P7, A5, O6), subjected to one-way ANOVA with Tukey’s multiple comparisons test. **f**, Transmission electron micrograph of protruding epithelial cell type, heavily burdened with SARS-CoV-2 virions (red) at 72 h p.i. Scale bar, 2 μm. **g**, Representative images of immunofluorescence staining for cells that have escaped the pseudostratified position and reside above the apical membrane, as stained in **a**. Of note here: SARS-CoV-2 spike (yellow) and KRT5 (white). Image 3D-rendered (left) using Imaris (Bitplane) with Blend filter; scale bar, 60 µm. Scale bar for all other images: 5 µm, rendered in ImageJ in right bottom panel, showing a histogram of distance vs fluorescence intensity for DAPI, KRT5 and SARS-CoV-2 spike staining for a single *Z*-slice indicated by purple dotted line. **h**, Transmission electron micrograph of epithelial cell shedding (white arrows) at 72 h p.i. with SARS-CoV-2. **i**,**j**, UMAP representation of the results from Milo differential abundance (DA) testing (left plot) with nodes showing cell neighbourhoods and Beeswarm plot (right plot) showing the log(FC) observed when comparing SARS-CoV-2-infected versus mock conditions in paediatric **i**, and older adults **j**, with a significant enrichment of goblet 2 inflammatory cells and basaloid-like 2 cells, respectively, observed with infection. Beeswarm plot shows the distribution of log(fold change) across annotated cell clusters when comparing SARS versus mock groups, with cell types ranked on the basis of those with the highest fold change. Grey is non-significant, red is significantly increased, blue is significantly decreased at 10% FDR. **k**, UMAP visualization of annotated epithelial cells from lower and upper airways of 8 in vivo integrated single-cell datasets. Cell numbers per cell type are shown in parentheses. **l**,**m**, Graph comparing the frequency of (**l**) goblet inflammatory and (**m**) basaloid-like 2 cells normalized to the total number of cells per donor. Each dot represents the ratio of the number of cells multiplied by 1,000 to the total cells contributed from one donor and are coloured on the basis of age_status group. Healthy dataset *n* = P49, A45, O46; COVID-19 dataset *n* = P41, A58, O116. Statistical analysis was performed on the normalized proportions using zero-inflated Poisson models using the gamlss package in R. Boxplots show the median and IQR, plus the minimum and maximum value distribution. Note the large frequency of donors with zero incidence. Source data

**Extended Data Fig. 4:**
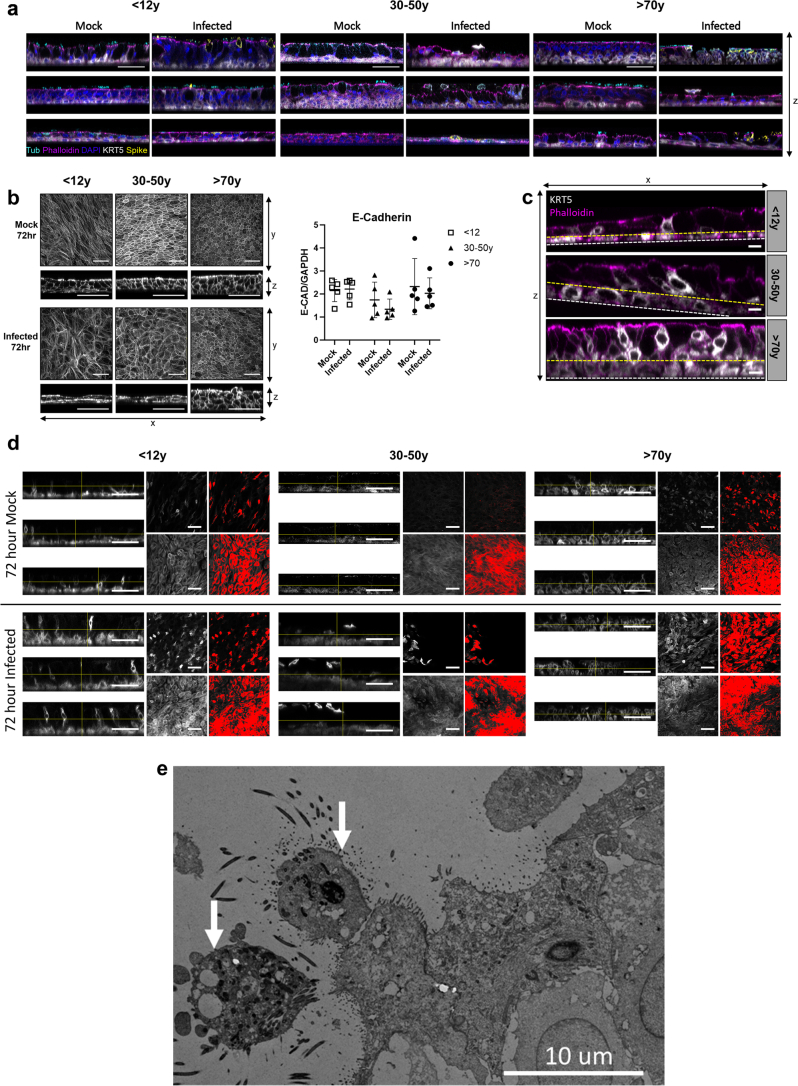
Minimal cytopathology following SARS-CoV-2 infection of NECs. **(a)** Orthogonal views of the z-stacks showing the thickness and morphology from fixed paediatric, adult and older adult NECs from 3 separate donors at 72 h p.i. mock or SARS-CoV-2 infected NEC cultures. Sections were immunolabeled against cilia (tubulin, cyan), F-actin (phalloidin, magenta) DAPI (blue), SARS-CoV-2 S protein (yellow) and cytokeratin 5 (KRT5+, white). Scale bar = 50 µm. **(b)** Representative maximal intensity projections (top panels) and orthogonal z sections (bottom panels) of NECs stained for E-Cadherin at 72 h p.i. with mock or SARS-CoV-2 (scale bar 50 μm) and expression determined via Western blots and normalised to GAPDH. Data plotted as mean ± SD, subject to one-way ANOVA with Tukey’s multiple comparison test, with individual values shown (n = P5, A5, O5). **(c)** Representative orthogonal views of the z-stacks showing the localisation of KRT5+ve cells (white) from example NEC cultures (summary in Fig. 2d). KRT5+ve cells above the horizontal yellow line are classified as non-basal -layer (not in contact with basement membrane), F-actin (phalloidin, magenta) stain references apical membrane and tight-junctions. Each section is 225 µm in width. **(d)** Maximal projections of the z-stacks showing the localisation of KRT5+ve cells (white) from above and below the horizontal yellow line (classified as non-basal -layer) from example air–liquid interface cultures at 72 h p.i. with mock or SARS-CoV-2 (scale bar 50 μm). **(e)** Transmission electron micrograph of epithelial cell shedding (white arrows) at 72 h p.i. with SARS-CoV-2 (scale bar 10 μm). Source data

**Extended Data Fig. 5:**
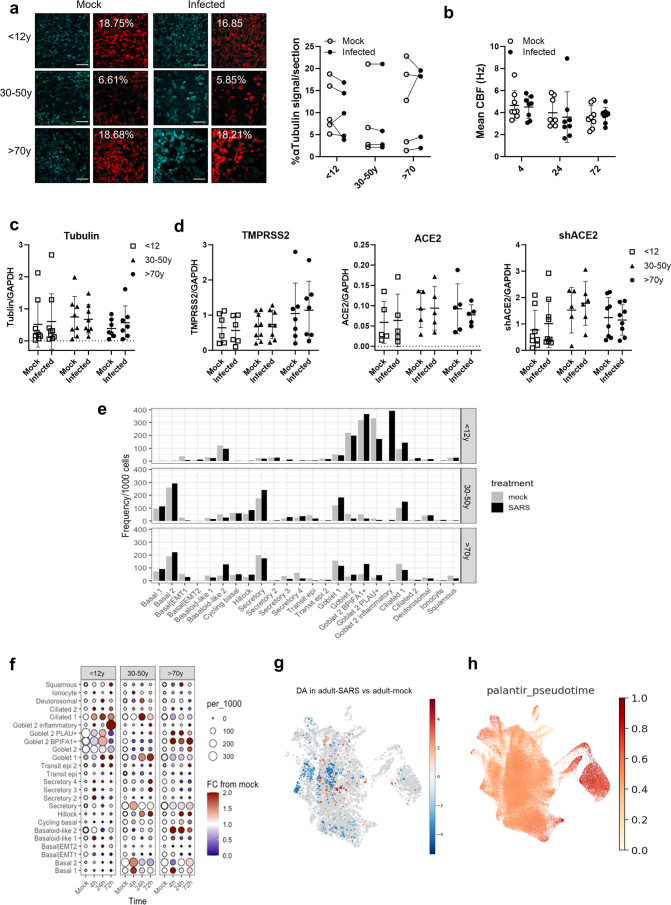
Cytopathology and changes in cell types. **(a)** Cilia coverage for each age group at 72 h p.i. Mock and SARS-CoV-2. (left) Representative Immunofluorescent images from 72 h p.i. NECs with mock and SARS-CoV-2 condition stained for a-tubulin (cyan) (scale bar = 50 µm). Percentage area covered (right) with αTubulin+ve signal (cyan) from maximum intensity projections of fixed NECs using threshold analysis (red) in ImageJ. Summary of cilia coverage (right) (n = P5, A4, O5). Subject to one-way ANOVA with Tukey’s multiple comparison test. For 72 h p.i. (mock and SARS-CoV-2 conditions) NECs were compared between age groups, looking at average **(b)** cilia beat frequency (Hz) (n = P8, A8, O8); **(c)** α-Tubulin protein expression (n = P11, A8, O7) and **(d)** SARS-CoV-2 entry factor protein expression (ACE2, TMPRSS2 and short ACE2). Protein levels were determined via Western blot and normalised to GAPDH (n = P6-8, A5-9, O5-8). Data for b-d plotted as mean ± SD, with individual values shown. **(e)** Total numbers of annotated airway epithelial cells in all mock infected vs all SARS-CoV-2 infected datasets in respect to age (data shown in ratio of cell numbers/per 1000 cells in age dataset) (n = P3, A4, O4). **(f)** Dot plots showing the log-fold change in SARS-CoV-2 versus mock at different time points for all cell types in different age groups. Calculation based upon absolute cell numbers with the average fold-change indicated by colour. The dot size corresponds to the number of that cell type per 1000 cells from each condition. **(g)** Uniform manifold approximation and projection (UMAP) representation of the results from Milo differential abundance testing. Nodes are neighbourhoods, coloured by their log fold change when comparing SARS-CoV-2 infected versus mock conditions in adult samples. Non-significant DA neighbourhoods at FDR 10% are coloured grey and significant DA neighbourhoods at FDR 10% are coloured with increased log fold change in red and decreased log fold change in blue. Node sizes correspond to the number of cells in a neighbourhood. The layout of nodes is determined by the position of the neighbourhood index cell in the UMAP. **(h)** Palantir inferred pseudotime probabilities of cycling basal cells differentiating into ciliated 1, basaloid-like 2 or goblet 2 inflammatory cells. Source data

Overall, we found that compared to uninfected cultures, SARS-CoV-2-infected adult (*P* < 0.05, *n* = 5) and older adult (*P* < 0.001, *n* = 7) cultures had decreased culture thickness (Fig. 2a,b and Extended Data Fig. 4a) and epithelial integrity (*P* < 0.03, *n* = 7; Fig. 2c), with no change in adherens junction protein expression (Extended Data Fig. 4b). This decrease in culture thickness was accompanied by an increase in basal cell mobilization (*P* < 0.03, *n* = 7; Fig. 2d and Extended Data Fig. 4c,d) and epithelial escape (cell protrusion) from the pseudostratified culture in older adult cultures (Fig. 2a,e). Some protruded cells carried viral particles (Fig. 2f) and expressed the SARS-CoV-2 spike protein (Fig. 2g) and others were shown to completely detach from the pseudostratified epithelium on the apical surface of the culture (Fig. 2h and Extended Data Fig. 4e). Ultrastructural changes such as endocytosis of cilia basal bodies and sloughing of ciliated cells were observed in all age groups (Supplementary Fig. 3). However, there was no significant loss of ciliated cells or changes in ciliary beat frequency (Extended Data Fig. 5a–c), or entry factor protein expression within 72 h of infection (Extended Data Fig. 5d).

**Extended Data Fig. 6:**
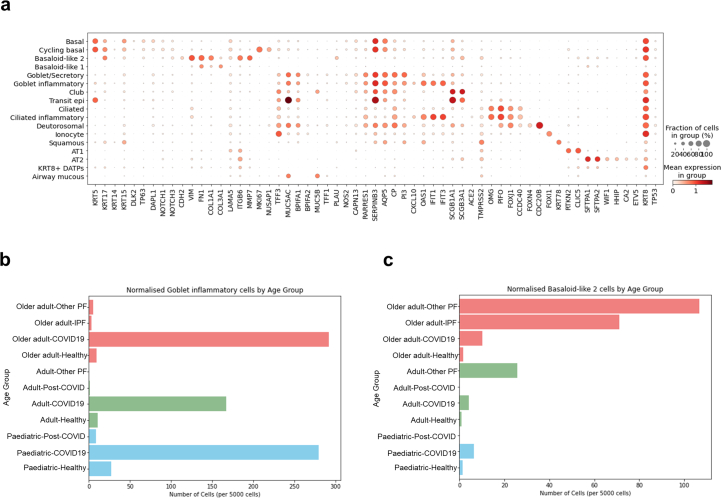
Basaloid-like cells were predominantly found in COVID-19 older adult patients *in vivo.* **(a)** Dot plot visualisation showing marker genes for annotated epithelial cell types in the integrated *in vivo* single cell dataset (Fig. 2k), with fraction of expressing cells and average expression within each cell type indicated by dot size and colour, respectively. Normalised goblet inflammatory cells **(b)** and basaloid-like 2 cells **(c)** per total of 5000 cells per age group in all paediatric, adult, and older adult subgroups. PF = pulmonary fibrosis, IPF = idiopathic pulmonary fibrosis. (Healthy dataset n = P49, A45, O46; COVID19 dataset n = P41, A58, O116). Source data

Using Milo^14^, we tested for differential cell state abundance following infection and whether this varied with age. In paediatric cultures, the most significant change was the emergence of goblet 2 inflammatory cells, which were not present in uninfected paediatric cultures (Fig. 2i and Extended Data Fig. 5e,f). There was a decrease in basal, secretory and goblet cell populations, while the frequency of transit epi 2 and terminally differentiated goblet cells increased (that is, goblet 2 inflammatory) (Fig. 2i and Extended Data Fig. 6e,f).

The goblet 2 inflammatory cell type is strongly associated with type I IFN signalling, with higher levels of *CXCL10*, *IFIT1* and *IFIT3* markers than other goblet cell subtypes (Extended Data Fig. 1d). While goblet inflammatory cells have previously been seen in vivo^9^, it is interesting that this inflammatory phenotype is epithelial cell-intrinsic and independent of immune cells that are not present in our cultures. We later (see next section) explore the impact of this on viral replication and spread.

The biggest and consistent change in infected older adult cultures was an increase in basal (*KRT5*^*hi*^) cell populations, indicating an older adult-specific mobilization (proliferation) of progenitor cells following SARS-CoV-2 infection (Fig. 2j, adult dataset shown in Extended Data Fig. 5f,g) and an expansion of basaloid-like 2 cells (Fig. 2j and Extended Data Fig. 5f). These recently identified cells are characterized by markers associated with tissue injury and fibrosis (*ITGB6*, *ITGB1*, *ITGAV*, *ITGB8*, *VIM*, *TGFB1*) (Extended Data Fig. 1d). In healthy epithelial tissue, including skin and lung, integrin beta 6 (*ITGB6*) mRNA is virtually undetectable^15^, but its expression has been reported to be considerably upregulated during wound healing^16^, tumorigenesis and fibrosis^17^. The presence of these *ITGB6*+ cells is a major finding as they may be involved in the exacerbation of disease in older adults.

Pseudotime trajectory analysis suggested that goblet 2 inflammatory cells (Extended Data Fig. 2h) and basaloid-like 1 cells (Extended Data Fig. 2j) are terminal cell states, differentiating from goblet 2 PLAU+ (Extended Data Fig. 2i) and Basal|EMT cells (Extended Data Fig. 2k), respectively, with ciliated 1 cells seen as a third end state (Extended Data Fig. 5h)

### In vivo patient validation of induced cell states

To confirm the existence of deregulated cell states in vivo, we performed an integration of 8 scRNA-seq datasets comprising 577,243 cells, spanning upper and lower airways from paediatric (0–18 years), adult (19–50 years), and older adults (51–90 years) that are either healthy or COVID-19 patients^9,10,12,18–22^ (Fig. 2k and Extended Data Fig. 6a–c). We identified common epithelial clusters by marker genes (Extended Data Fig. 6c). Goblet inflammatory cells were induced in response to SARS-CoV-2 across all age groups, with the highest abundances in paediatric COVID-19 and older adult COVID-19 cohorts (Fig. 2l and Extended Data Fig. 6b). We note that in the older adult COVID-19 cohort, a single donor (mild disease, early post-symptom samples) contributed 82% of all goblet inflammatory cells (Fig. 2l). Thus, the induction of this cluster is most robust in the paediatric cohort. In the in vivo dataset, we also identified a basaloid-like 2 cell cluster enriched across all COVID-19 patients, which were most abundant in older adult COVID-19 patients across multiple donors (Fig. 2m and Extended Data Fig. 6c), confirming our in vitro studies. Basaloid-like 2 cells also had the highest increase in fibrosis patients (both idiopathic pulmonary fibrosis and other pulmonary fibrosis), as previously reported^11,23^ (Extended Data Fig. 6c).

### Stronger interferon response in paediatric cultures

**Fig. 3:**
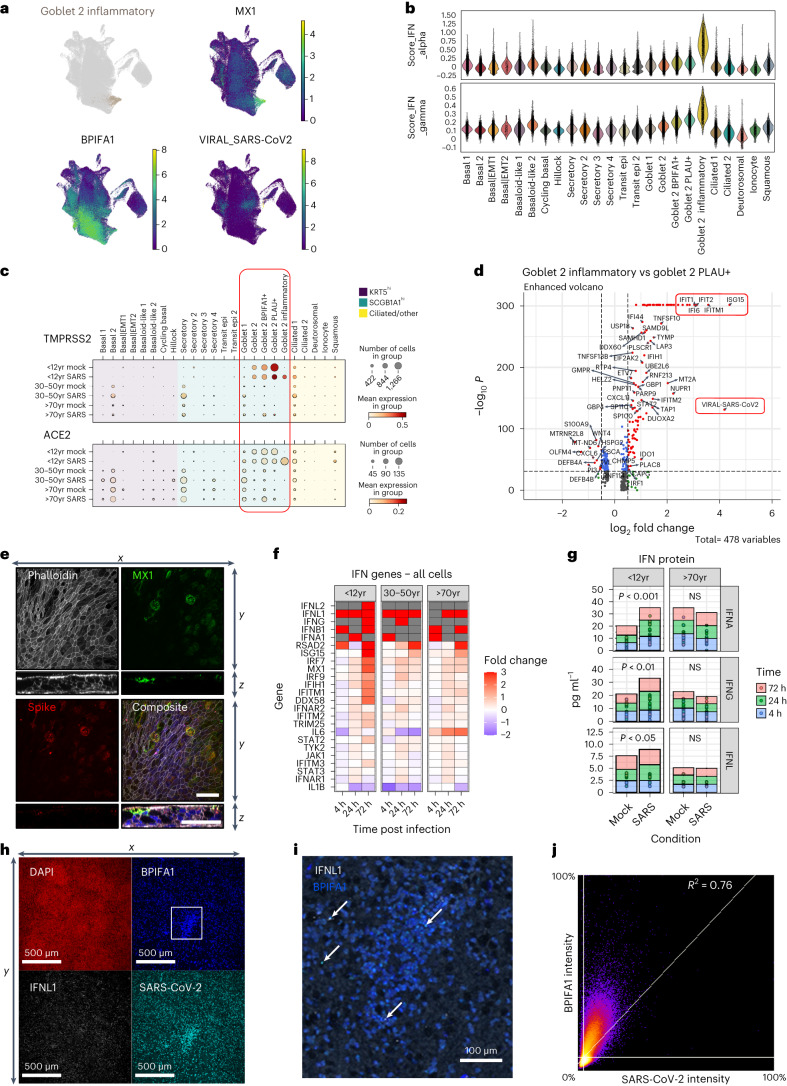
Paediatric goblet 2 inflammatory cells and interferon expression. **a**, UMAP visualization of expression of differentially expressed genes in goblet 2 inflammatory cells. Gene expression is shown in log1p scale. **b**, Scores of gene ontology (GO) term gene signatures for the terms: response to type 1 interferon (GO:0035455 or GO:0034340) and type 2 interferon (GO:0034341) across cell types. Scores were calculated with Scanpy as the average expression of the signature genes subtracted with the average expression of randomly selected genes from bins of corresponding expression values. Each dot is a cell. **c**, SARS-CoV-2 entry factor gene expression per cell type calculated on the basis of absolute cell numbers with the average expression of *TMPRSS2* (top) and *ACE2* (bottom) indicated by colour. Dot size corresponds to infected number of cells expressing *TMPRSS2* and *ACE2* in respective age groups in the mock (all timepoints) and SARS-CoV-2 (all timepoints) infected condition. **d**, Volcano plot showing differential gene expression between goblet 2 inflammatory and their precursor goblet 2 PLAU+ cells, with a total of 478 variables. Of note were several genes associated with an interferon response (for example, *IFI6*, *IFITM1*, *IFIT1*, *IFIT2* and *ISG15*) and SARS-CoV-2 viral replication (highlighted in red) which were significantly enriched within the paediatric goblet 2 inflammatory cells. The colours indicate the genes that have adjusted *P* values ≤0.05 (blue), a log_2_ fold-change ≥1 or ≤−1 (green), or remain unchanged (grey). The dashed horizontal line signals statistical significance threshold (adjusted *P* values ≤0.05). Two vertical lines show the threshold of log_2_ fold-change ≥0.5 and ≤−0.5. **e**, Visualization of MX1 protein-expressing cells. Maximum intensity projection images of immunofluorescence staining for F-actin (white, phalloidin), MX1 (green), SARS-CoV-2 S protein (red), with DAPI (blue) in composite image. An orthogonal view of the *z*-stacks is given in the bottom panel. Example given is a SARS-CoV-2-infected paediatric culture at 72 h p.i. Scale bar, 50 µm. **f**, Fold change in the gene expression in selected IFN genes across all cell types in SARS-CoV-2-infected NECs compared to mock infections in the single-cell datasets. Shown at each timepoint and broken down by age group. Where no expression was seen in the mock infection conditions, fold change was capped at 3 (red). Grey highlights genes that were absent in both conditions. **g**, Level (pg ml^−1^) of interferon protein (IFNA, IFNG and IFNL) within the apical supernatant between SARS-CoV-2 and mock-infected NECs. Two-way paired *t*-test. *P = 0.05, **P < 0.01. (*n* = P9, O9). **h**, Representative immunofluorescence images of inflammatory goblet cell markers at 72 h p.i. with SARS-CoV-2. Maximum intensity projection images of immunofluorescence staining in fixed paediatric NECs. Red, DAPI; white, IFNL1; blue, BPIFA1; cyan, SARS-CoV-2 spike (S) protein. **i**, Higher magnification image of that shown in **h** with white IFNL1; blue, BPIFA1 (white arrows annotate inflammatory goblet cells). **j**, Co-localization plot for BPIFA1 and SARS-CoV-2 S protein. Source data

**Extended Data Fig. 7:**
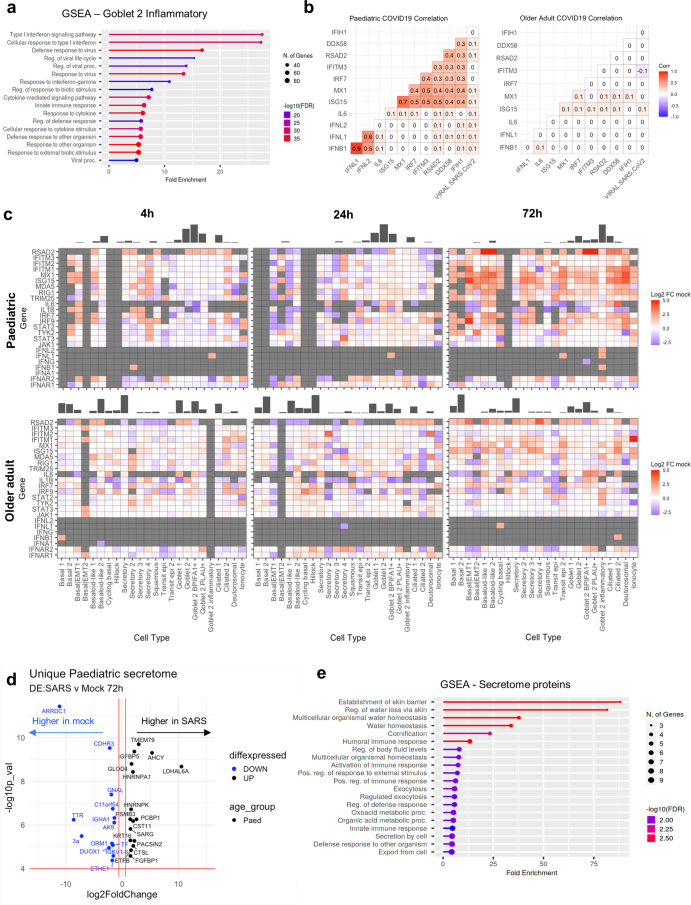
Paediatric goblet 2 inflammatory cells and viral truncation in response to IFN signalling. **(a)** Gene Set Enrichment Analysis (GSEA) of HVGs from goblet inflammatory cells indicating enriched gene ontology terms using ShinyGo. **(b)** Correlation matrix of a subset of interferon genes expressed by all paediatric or older adult cells at 72 h post SARS-CoV-2 infection as determined using the function cor_pmat() in ggcorrplot and Pearson correlation method. **(c)** ISG gene expression in SARS-CoV-2 infected cultures (as log-fold change compared to mock) per cell type at 4 h, 24 and 72 h p.i. for paediatric and older adult datasets. Barplot indicates the number of cells at each time point. Grey = not detected **(d)** Volcano plot showing differential expressed proteins of the apical secretome between mock and SARS-CoV-2 infected cultures that were unique (highly expressed) in the paediatric cohort. Blue highlights those that are highly expressed in mock compared to SARS-CoV-2 infection conditions and black enriched with infection. **(e)** GSEA for expression of apical secretome genes of paediatric cells at 72 h p.i. with SARS-CoV-2 obtained using ShinyGo. The data in **a,b,c** relates the scRNAseq dataset (n = P3, A4, O4), whilst **d** and **e** uses data generated through the analysis of the collected apical secretome (n = P5, A5, O5). Source data

As described, SARS-CoV-2 infection is associated with strong interferon responses, which were particularly apparent in paediatric goblet 2 inflammatory NECs but absent in mock-infected cultures and rare in infected older age groups (proportion of total goblet 2 inflammatory cells from NEC cultures: paediatric = 1,455/1,578, adult = 90/1,578, older adult = 33/1,578) (Extended Data Fig. 1g). These cells exhibited high levels of interferon-stimulated genes (ISGs), associated with both type I and II interferon signalling (Fig. 3a,b and Extended Data Fig. 7a,b), and were previously shown to reduce COVID-19 severity^10,24^. In addition, paediatric cultures-secreted proteins also showed an association with epithelial barrier and humoral immune response pathways (Extended Data Fig. 7c–e).

The precursors of goblet inflammatory cells are goblet 2 PLAU+ cells (Extended Data Fig. 2i) which expressed high levels of *TMPRSS2* and *ACE2* (Fig. 3c), suggesting that the virus targeted these cells and induced the generation of goblet inflammatory cells, which again expressed high levels of entry receptors and could thus be the target for further infection. This is supported by high viral reads and high ISG expression specific to this subtype (Fig. 3d). Coexpression of viral spike protein and the interferon-induced gene *MX1* was confirmed at the protein level in our paediatric cultures (Fig. 3e). Induction of interferon responsive genes appears to be at least partly autocrine since paediatric inflammatory cells transcribed *IFNL1*, *IFNL2* and *IFNA1* genes (Fig. 3f and Extended Data Fig. 7c). When comparing the ISG response across all cell types and ages, it is apparent that by 72 h p.i. paediatric cultures express more interferon genes (Fig. 3f), a difference that was validated at the protein level (Fig. 3g). Furthermore, immunofluorescence staining demonstrated the co-localization (*R*^2^ = 0.76) of IFNL1 with the goblet 2 inflammatory cell marker BPIFA1 (Fig. 3h,i).

### Goblet inflammatory cells may restrict viral replication

**Fig. 4:**
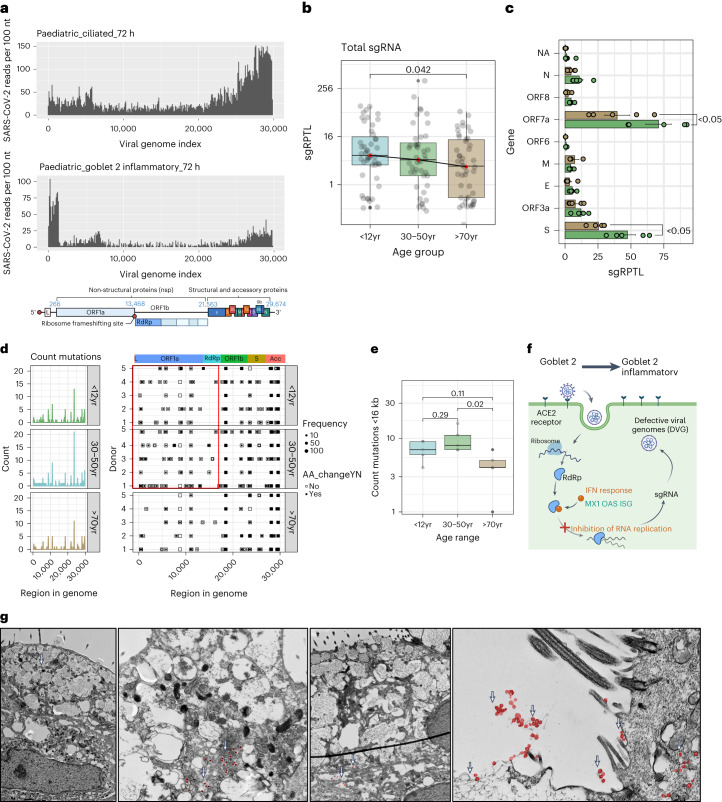
Incomplete viral replication in response to IFN signalling in paediatric goblet 2 inflammatory cells. **a**, Coverage plot of viral reads aligned to SARS-CoV-2 genome from paediatric ciliated 2 (top) and goblet 2 inflammatory (middle) cells at 72 h p.i. Bottom panel shows the genomic organization of SARS-CoV-2 as drawn using Biorender.com. The sequencing depth was computed for each genomic position for each condition. **b**, Boxplot depicting the sgRPTL normalized counts for sgRNA abundances across age groups using unpaired *t*-test. **c**, The mean ± s.d. distribution of these sgRPTL counts across all genes in paediatric (green) and older adult (brown) NEC cultures, subjected to two-way ANOVA with Sidak’s multiple comparisons test (*n* = P5, O5). **d**, Left: frequency of genomic mutations observed in different regions of the SARS-CoV-2 genome. Right: the position and whether an amino acid change was generated from that mutation. Data were generated from 72 h p.i. with SARS-CoV-2 (*n* = P5, A5, O5). Bin size is 50 bases. Colour blocks indicate the start coordinates of annotated viral genes. **e**, Number of genomic mutations occurring <16 kb in genome, shown by age group. Data generated from *n* = P5, A5, O5. **f**, Hypothesis of SARS-CoV-2-infected goblet 2 PLAU+ cells becoming protective goblet 2 inflammatory cells through increased interferon and defective viral genome production. Drawn using Biorender.com. **g**, Transmission electron micrographs of goblet cells at 72 h p.i. with SARS-CoV-2 at different magnifications. Scale bar, 2 μm. Viral particles are false-coloured in red and indicated with white arrows. Lines in box and whisker plots (**b**,**e**) indicate median, IQR and minimum to maximum of the distribution, with individual values for each cell (**b**) or NEC culture (**e**) shown. Source data

**Extended Data Fig. 8:**
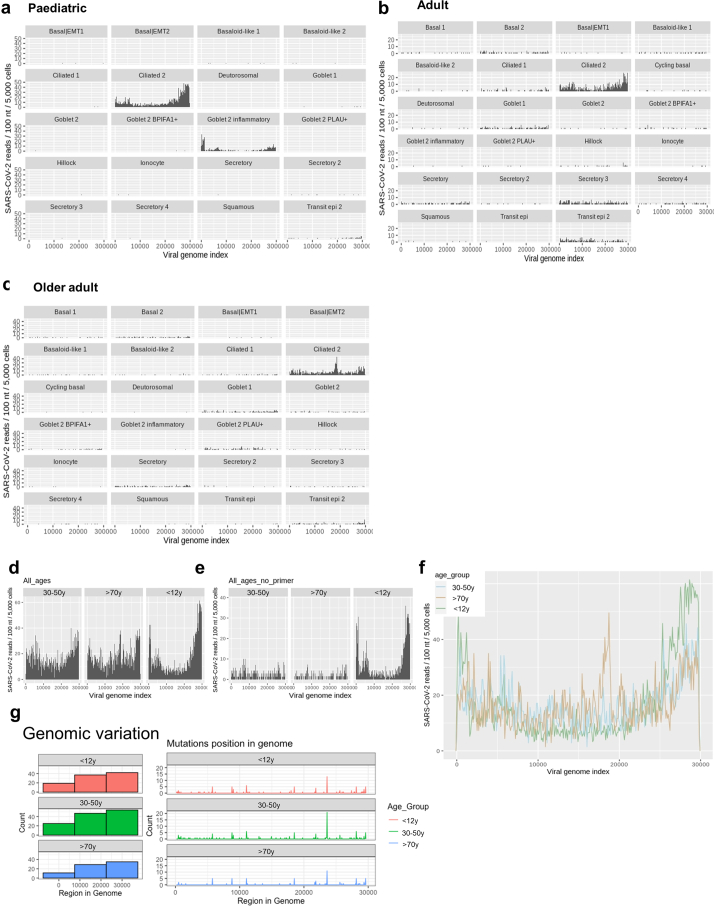
Viral truncation in response to IFN signaling. Coverage plots of viral reads aligned to SARS-CoV-2 genome in each cell type in **(a)** paediatric, **(b)** adult **(c)** and older adult infected NECs grouped across all time points. Coverage plots of viral reads aligned to SARS-CoV-2 genome for all cell types, shown by age group, both **(d)** with and **(e)** without spiked-in primer grouped across all timepoints (see methods for more details of primer) and at **(f)** 72 h p.i. time points. Viral reads for all coverage plots are shown in 100 nucleotide (nt) bins normalised per 5,000 cells. The data in **a**-**f** relates to the scRNA-seq dataset (n = P3, A4, O4). **(g)** Histogram displaying frequency and position of genomic mutations in SARS-CoV-2 consensus sequences from 72 h p.i. with SARS-CoV-2 (n = P5, A5, O5). Bin size is 1000 bases (left) and 50 bases (right). Colour blocks indicate the start coordinates of annotated viral genes. Source data

In paediatric cultures, despite high viral reads, the production of infectious virions is lower than in older adult cultures (Fig. 1l). Examining the distribution of viral reads, we found that viral transcription in paediatric ciliated cells predominantly occurred towards the 3’ end, indicating active viral replication (Fig. 4a). However, in paediatric goblet 2 inflammatory cells, viral reads were highest near the 5’ end, suggesting failed viral replication (Fig. 4a and Extended Data Fig. 8a–c). It was concluded that this bias towards the 3’ end was not a technical artefact due to the introduction of the spike-in primer to increase the detection of viral reads, as SARS-CoV-2 reads were successfully amplified without biasing viral distribution (Extended Data Fig. 8d–f). Moreover, using deep viral sequencing, we found that non-canonical subgenomic SARS-CoV-2 RNAs (sgRNA), particularly spike and ORF7a sgRNA, were more abundant (*P* = 0.042) in paediatric and adult samples than in older adult samples (Fig. 4b,c). These non-canonical sgRNAs can result in defective viral genomes and have been associated with increased interferon production^25^. Paediatric cultures also exhibited more low-frequency and fixed mutations in viral genomes (Fig. 4d and Extended Data Fig. 8g), particularly before the RNA-dependent RNA polymerase (RdRp) (that is, <16 kb; Fig. 4e). These findings suggest that there is greater pressure on the virus to mutate in younger cultures, possibly due to the production of defective viral genomes by goblet 2 inflammatory cells (Fig. 4f). In addition, ultrastructural observations revealed fewer viral particles in paediatric goblet cells than in heavily burdened neighbouring ciliated cells (Fig. 4g and Supplementary Fig. 2). Our findings indicate that paediatric goblet inflammatory cells may be responsible for the discrepancy between viral reads and infectious particles.

### Infected older adult cultures express pro-fibrotic and EMT markers

**Fig. 5:**
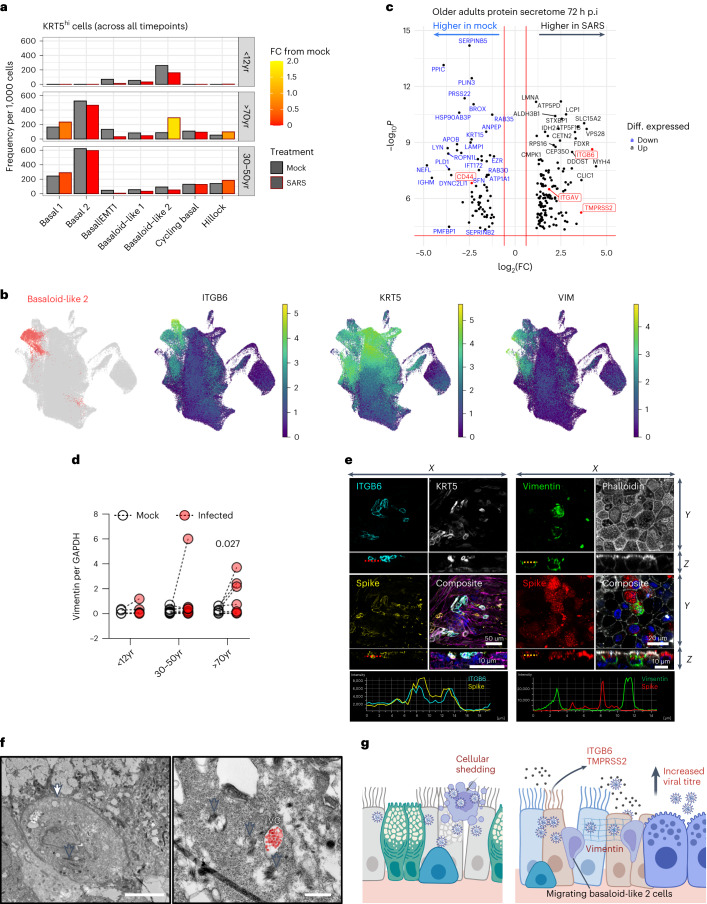
Elderly basaloid-like 2 cells drive ITGB6 production and enhance viral pathogenesis. **a**, Frequency of KRT5^hi^ basal airway epithelial cells in mock (black outline) and SARS-CoV-2-infected (red outline) conditions across all timepoints (4, 24 and 72 h p.i.) with respect to age. Data shown in ratio of cell numbers per 1,000 cells per age group within scRNA-seq dataset, where the colour of the bars indicates fold change (FC) from the matched cell compartment in the mock condition. **b**, UMAP visualization of expression of differentially expressed genes (*ITGB6*, *KRT5* and *Vimentin (VIM)*) in basaloid-like 2 cells. Gene expression is shown in log1p scale. **c**, Volcano plot of differentially expressed proteins in the apical secretome of mock- and SARS-CoV-2-infected cultures that were unique (highly expressed) in the older adult dataset. Blue highlights those that are highly expressed in mock compared with SARS-CoV-2 infection conditions and black are enriched with infection; of note: ITGAV, ITGB6 and TMPRSS2 in red. The red horizontal line signals statistical significance threshold (adjusted *P* values ≤0.05). Two vertical lines show the threshold of log_2_ fold-change ≥0.5 and ≤−0.5. **d**, Analysis of vimentin protein levels by Western blot normalized to GAPDH (*n* = P5, A9, O9), subjected to multiple paired ratio *t*-test. **e**, Representative immunofluorescence images of basaloid-like 2 cell markers in older adults at 72 h p.i. with SARS-CoV-2. Maximum intensity projection images of immunofluorescence staining in fixed older adult NECs. Left: cyan, ITGB6; white, KRT5; yellow, SARS-CoV-2 spike protein; and composite with F-actin (magenta, phalloidin) and DAPI (blue). Right: green, vimentin; F-actin (grey, phalloidin); red, SARS-CoV-2 S protein; and composite with DAPI (blue). White arrows annotate the vimentin cage structure around SARS-CoV-2 S protein. **f**, Transmission electron micrograph of migrating basal KRT5+ epithelial cell in older adult cultures at 72 h p.i. with SARS-CoV-2 (white arrow). Cytokeratin bundles are indicated (grey arrows) and viral compartments (VC) containing viral particles false-coloured in red. Scale bars, 5 μm (left) and 0.5 μm (right). **g**, Hypothesis that infection of older adult cells leads to increased shedding of cells heavily burdened with viral particles, which may result in further spread of infection. Repair processes increase KRT5+ and ITGB6+ basaloid-like 2 cells, which are prioritized over the early antiviral responses from goblet 2 inflammatory cells, thereby elevating viral titre. Drawn using Biorender.com. Source data

**Extended Data Fig. 9:**
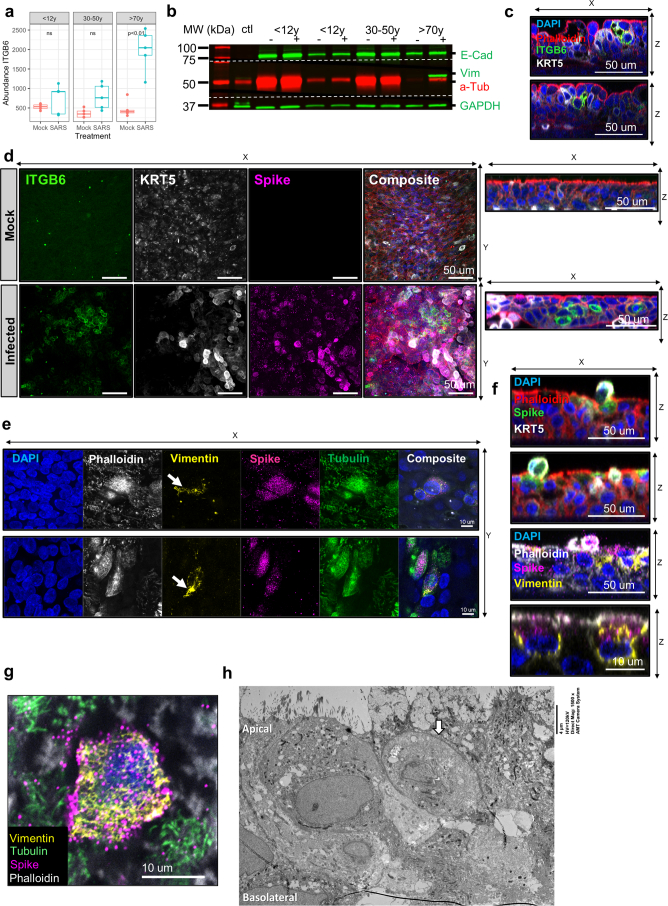
Elderly basaloid-like 2 cells drive ITGB6 production and enhance viral pathogenesis. **(a)** Abundance of ITGB6 protein in the apical secretome of mock or SARS-CoV-2 infected NECs at 72 h p.i. for each age group (n = P5, A5, O5). As detected using mass spectrometry and shown in boxplot depicting the median and IQR, plus the minimum and maximum value distribution analysed using paired t test. **(b)** Exemplar Western blot showing vimentin (vim) in the older adult sample 72 h p.i. with mock (-) or SARS-CoV-2 (+) and 72 h p.i. Vero E6 cells as control lysate (ctl). GAPDH is the loading control, E-Cadherin is also given for reference (n = P2, A1, O1). **(c)** Orthogonal views of the z-stacks showing the location of ITGB6 (green) and KRT5+ve cells (white) from exemplar air–liquid interface cultures, counterstained for F-actin (phalloidin, red) and cell nucleus (DAPI, blue). **(d)** Representative maximum intensity projections (left) and orthogonal sections (right) of immunofluorescence z-stacks of basaloid-like 2 cell markers ITGB6 (green), KRT5 (white), spike (magenta) counterstained for F-actin (phalloidin; red) and cell nucleus (DAPI, blue) in 72 h p.i. NECs (mock top, infected bottom). **(e-g)** Further, example immunofluorescence images of basaloid-like 2 cell markers in 72 h p.i. with SARS-CoV-2 in older adult NEC cultures. Markers and respective counterstain colour are indicated (n = O3). **(h)** Transmission electron micrograph of non-basal KRT5+ve epithelial cell (white arrow) location within an NEC culture at 72 h p.i. with SARS-CoV-2. Scale is on the right of the image. For panels **c-h**, representative images were selected from older adult NEC cultures (n = 3) 72 hours post-infection (p.i.) with SARS-CoV for each antibody panel. Source data

As discussed above, SARS-CoV-2 infection in older adult cultures led to an increase in basaloid-like 2 cells (Fig. 5a) associated with a pro-fibrotic state and epithelial–mesenchymal transition (EMT), including expression of *ITGB6*, *VIM* and *KRT5* (Fig. 5b and Extended Data Fig. 1d). Typically membrane bound proteins such as ITGB6, ITGAV and TMPRSS2, produced by these cells, were more abundant in the supernatant of infected cultures from older adults (Fig. 5c and Extended Data Fig. 9a), possibly originating from shed cells or debris. Vimentin (VIM) was upregulated in cell lysates of SARS-CoV-2-infected older adult cultures compared with mock (*n* = 9; *P* < 0.05) (Fig. 5d and Extended Data Fig. 9b). Immunofluorescence microscopy revealed the co-localization of ITGB6 protein with SARS-CoV-2 S protein (Fig. 5e and Extended Data Fig. 9c,d) and the formation of vimentin cages around the virus in some infected older adult cells (Fig. 5e and Extended Data Fig. 9e–g)^26^. Rare instances of migrating basal cell types (defined by the presence of cytokeratin bundles) burdened with viral compartments were also observed, suggesting that KRT5+, ITGB6+ and VIM+ cells are permissive to SARS-CoV-2 infection (Fig. 5f and Extended Data Fig. 9h).

Although basaloid-like 2 cells showed low levels of viral transcription (Fig. 1g), the most severely infected and damaged cells are probably shed into the secretome (as hypothesized in Fig. 5h), leading to more ITGB6 protein (Fig. 5c) and protruding cells (precursors of shed cells) in infected older adult cultures (Fig. 2e).

### ITGB6 expression in repair enhances viral replication

**Fig. 6:**
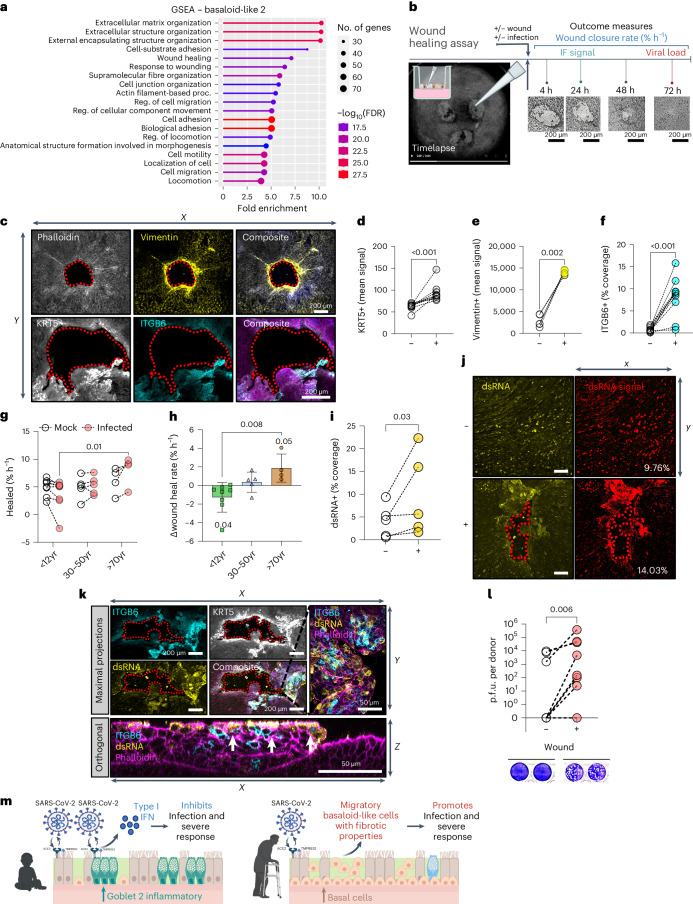
Wound healing upregulates basaloid-like 2 cell markers and associates with increased viral spread. **a**, GSEA indicating enriched gene ontology terms for basaloid-like 2 cells obtained using ShinyGo. **b**, Schematic to show the different wound healing assay protocols. **c**, Representative immunofluorescence images of basaloid-like 2 cell markers at 24 h post-wound NECs. Top: maximum intensity projection images (left to right): F-actin (grey, phalloidin); yellow, vimentin; and composite with DAPI (blue). Bottom (left to right): white, KRT5; cyan, ITGB6; and composite with F-actin (magenta, phalloidin) and DAPI (blue). Scale bar, 200 µm. Basaloid-like 2 cell markers mean fluorescence signal around wound area. Wound area shown by dotted red outline. **d**–**f**, Analysis of maximal intensity projections of fixed NECs without (−) and with (+) wounds at 24 h post wounding. **d**, KRT5+ (mean) signal (*n* = 9; P3, A3, O3). **e**, Vimentin+ (mean) signal (*n* = 5; P2, A1, O2). **f**, ITGB6+ % coverage (*n* = 10; P4, A4, O2), subjected to ratio paired *t*-test. Wound healing rate in NECs from different age groups with mock or SARS-CoV-2 infection. **g**, Percentage wound closure (healed) per hour (% h^−1^), subjected to two-way ANOVA with Sidak’s multiple comparisons test (*n* = P8, A5, O4). **h**, The difference in wound closure per hour between mock and SARS-CoV-2-infected cells from the same donor. Mean ± s.d. (*n* = P8, A5, O4), subjected to one-way ANOVA with Tukey’s multiple comparisons test. Age variable shown as shape (triangles, adults; circles, paediatric). **i**, dsRNA coverage for NECs irrespective of age group at 72 h p.i. Determined by percentage area covered with dsRNA signal (yellow) from maximum intensity projections of fixed NECs, subjected to ratio paired *t*-test (*n* = 5; P2, A1, O2). **j**, Representative immunofluorescent images from 72 h p.i. NECs with SARS-CoV-2 without (top) and with (bottom) wounding stained for dsRNA (yellow). Percentage area covered (right) with dsRNA+ signal from maximum intensity projections of fixed NECs using threshold analysis (red) in ImageJ, with the percentage coverage given at the bottom right of each image. **k**, Representative immunofluorescence images of basaloid-like 2 cell markers ITGB6 (cyan), KRT5 (white), dsRNA (yellow) and F-actin (magenta, phalloidin) in SARS-CoV-2-infected NECs. Maximum intensity projection images from wounded cultures after 24 h, shown both as maximal projections (top) and as an orthogonal view (bottom). KRT5 (white) is omitted from composite images, so that overlap of ITGB6 (cyan) and dsRNA (yellow) is apparent (white). **l**, Infectious viral titres at 72 h p.i. in combined cell lysate and apical fluid of SARS-CoV-2 nasal epithelial cells from non-wounded (−) and wounded (+) donors that were previously shown to propagate low levels of infectious particles (<10,000 p.f.u. per donor at 72 h p.i.). Infectious viral load in combined apical and cell lysates (p.f.u. per donor) were determined by plaque assays, with representative plaque assay wells shown (bottom). Subjected to paired *t*-test (*n* = 8; P6, A2). **m**, Summary figure highlighting the key findings from the study. Created with BioRender.com. Source data

**Extended Data Fig. 10:**
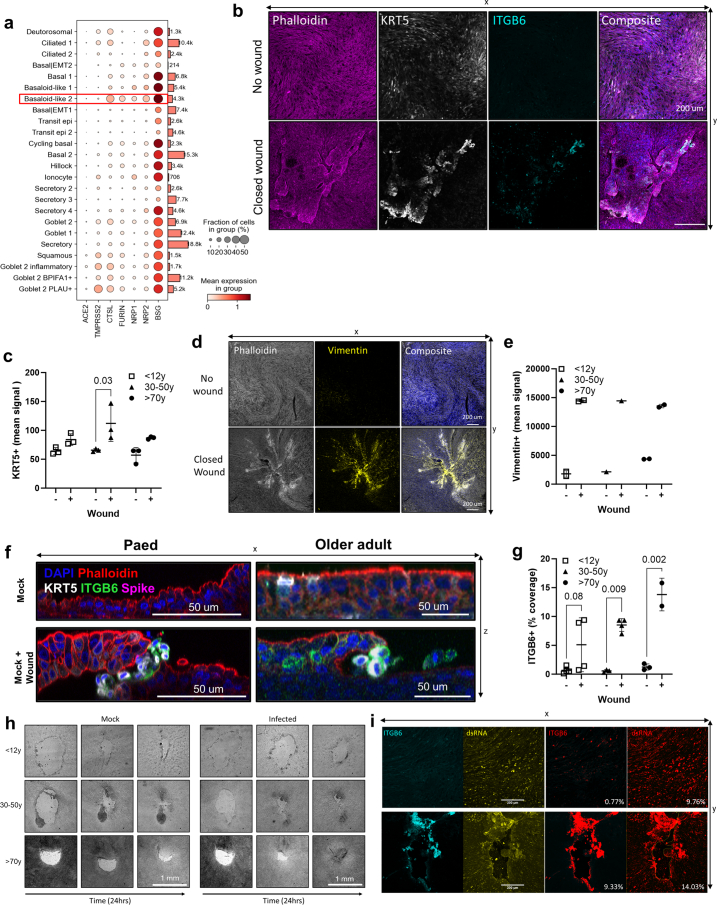
Wound repair promotes SARS-CoV-2 viral replication. **(a)** Dotplot visualisation showing viral entry genes for all cell types, with fraction of expressing cells and average expression within each cell type indicated by dot size and colour, respectively. Appended bar graphs indicate absolute cell numbers per cell type. Data generated using the entire scRNAseq dataset (n = P3, A4, O4). **(b)** Example immunofluorescence orthogonal sections of basaloid-like 2 cell markers in unwounded or wounded older adult NECs 24 h p.i. with mock or SARS-CoV-2 infection. F-actin (Phalloidin; red), KRT5 (white), ITGB6 (cyan) and DAPI (blue). **(c)** Mean ± SD KRT5 fluorescence signal (RFU) around wound area in different age groups. From maximal projections of fixed NECs without (-) and with (+) wounds, 24 h post wounding (n = P3, A3, O3) (n = 6). **(d)** Maximum intensity projection image of a NEC culture unwounded and 24 h post wound. F-actin (Phalloidin; white), Vimentin (VIM) (yellow) and DAPI (blue). **(e)** Mean ± SD Vimentin fluorescence signal (RFU) around wound area in different age groups. From maximal projections of fixed NECs without (-) and with (+) wounds, 24 h post wound (n = P2, A1, O2). **(f)** Example immunofluorescence orthogonal sections of basaloid-like 2 cell markers in non-wounded or wounded NECs 24 h p.i. with mock or SARS-CoV-2 infection from a paediatric and older adult donor. Stained for F-actin (Phalloidin; red), ITGB6 (green), SARS-CoV-2 Spike protein (magenta), KRT5 (white) and composite with DAPI (blue). **(g)** Mean ± SD ITGB6 fluorescence signal (RFU) around wound area in different age groups. From maximal projections of fixed NECs without (-) and with (+) wounds, 24 h post wounding (n = P3, A3-4, O3-2). **(h)** Example of wound healing images taken of NECs from different age groups and with mock or SARS-CoV-2 infection. Acquired by light microscopy over 24 hours. The scale bar (bottom right) represents 1 mm. **(i)** Representative immunofluorescence images from 72 h p.i. NECs with SARS-CoV-2 without (-) and with (+) wounding stained for ITGB6 (cyan) and dsRNA (yellow). Percentage area covered (right) with ITGB6+ve or dsRNA+ve signal from maximum intensity projections of fixed NECs using threshold analysis (red) in ImageJ, the percentage coverage is given at the bottom right of each image. For **c,e,g** the average values for each NEC donor are shown and are subject to a two-way ANOVA with Sidak’s multiple comparisons test. For panels **b, d, f, g, i** a minimum of 5 experiments (ranging from n = 5–10) were conducted for each antibody panel, from which representative images were selected. Source data

To investigate the role of basaloid-like 2 cells in SARS-CoV-2 pathogenesis, we performed gene set enrichment analysis (GSEA) and found that these cells are associated with extracellular matrix reorganization, wound response and migration processes (Fig. 6a). Such processes may facilitate viral spread, metastasis and fibrogenic remodelling^27–29^. They also showed upregulation of alternative viral entry receptors *CTSL*, *FURIN*, *NRP1* and *NRP2* (Extended Data Fig. 10a), suggesting their potential as targets for infection and spread.

To functionally observe these processes, we employed a wound healing assay (Fig. 6b). This assay enabled us to stimulate epithelial repair pathways, resulting in the increased expression of basaloid-like 2 cell markers around the wound site including KRT5 protein (Fig. 6c,d and Extended Data Fig. 10b,c) (mean signal ± s.d. 62.3 ± 8.40 to 94.3 ± 21.1; *P* < 0.001, *n* = 9), VIM (Fig. 6c,e and Extended Data Fig. 10d,e) (mean signal ± s.d. 2,887 ± 1,378 to 14,088 ± 518; *P* < 0.002, *n* = 5) and ITGB6 (Fig. 6c,f and Extended Data Fig. 10f,g) (%coverage ± s.d. 0.72 ± 0.05 to 8.13 ± 4.73; *P* < 0.001, *n* = 9). Although we found no difference in wound healing rate of uninfected cultures across ages (Extended Data Fig. 2c), SARS-CoV-2-infected older adult cultures exhibited a faster (*P* = 0.01) wound healing rate (% h^−1^, mean ± s.d. 8.01 ± 2.67% *n* = 4) compared with infected paediatric cultures (3.67 ± 2.78%, *n* = 8) (Fig. 6g,h and Extended Data Fig. 10h), indicating greater cell motility and altered repair processes in the older adult. Stimulating wound repair also correlated with an increase in SARS-CoV-2 infection, as evidenced by a higher percentage of dsRNA-positive cells (indicative of replicating virus) in wounded cultures (mean ± s.d. 4.09 ± 3.61% to 9.69 ± 9.04%; *P* = 0.03, *n* = 5) (Fig. 6i,j and Extended Data Fig. 10i), particularly around the site of the wound (Fig. 6k and Supplementary Fig. 1). Finally, cultures initially generating low infectious particle counts (<10,000 p.f.u. per donor at 72 h p.i.) showed increased viral particle production upon wounding (*P* = 0.006, *n* = 8) (Fig. 6l). These data suggest that basaloid-like 2 cells play a role in viral spread and their involvement in the wound healing process may contribute to SARS-CoV-2 infection and replication.

## Discussion

In our comprehensive study of SARS-CoV-2 infection in human NECs, we identified age-associated differences in COVID-19 pathogenesis. Key findings include the induction of a strong early interferon response in paediatric epithelial cultures infected by SARS-CoV-2, leading to incomplete viral replication. In contrast, NEC cultures derived from older adults produce more infectious viruses across various epithelial cell types when compared with paediatric cultures. Moreover, infected NECs from older adults exhibit increased cell shedding, thinning and leakiness, accompanied by the migration of basaloid-like 2 cells associated with wound repair. Interestingly, we showed that previous wounding of cultures resulted in an increased expression of basaloid-like 2 cell genes, promoting viral spread and ultimately augmenting infectious viral yield. Together, these findings contribute towards a deeper understanding of the age-specific nuances of the upper airway and the effects these may have on the pathogenic mechanism underlying SARS-CoV-2 infection across different ages.

Our in vitro model using primary NECs closely resembles SARS-CoV-2 infection in the human airway, the primary site of infection^30^. It confirms age-related changes in upper airway progenitor basal cell types reported previously^10,31^ and allows for the detection of intrinsic age-related differences in epithelial cells without confounding variations in host immunity.

Notably, we observed a significant shift in the *SCGB1A1*^*hi*^ cell population, transitioning from goblet cells in paediatric cultures to secretory cells with age, with the latter expressing higher levels of SARS-CoV-2 entry factors (ACE2 and TMPRSS2). In paediatric cultures, goblet 2 cell types were a primary target of infection, while in older adult cultures, infected secretory cells accounted for the highest proportion of viral reads.

Through the integration of existing in vivo COVID-19 datasets, we confirmed the existence of both basaloid-like 2 and goblet inflammatory cells identified in vitro to be induced in an age-dependent response to infection. This validates the early epithelial response to SARS-CoV-2 in our in vitro model. However, we acknowledge differences between the in vitro models and patient responses. For example, basaloid-like 2 cells are far less abundant in vivo, as has been reported^32^, which may be due to the site of sampling, cell dissociation protocols or other technical factors^33^. On the other hand, viral and cellular dynamics can be timed more precisely in vitro, while the time interval from the initial infection to sampling is largely unknown for in vivo studies as they are estimated from symptom onset.

We found that fewer paediatric cell types contained viral reads compared with adult and older adult cultures. This is consistent with previous studies indicating that infection in paediatric cells is confined to a limited number of cells due to an early interferon response that limits viral spread^34^. We suggest that this effect is attributed to goblet 2 inflammatory epithelial cells, which decrease with age. These cells have the highest viral genome burden and the strongest interferon signature of all epithelial subtypes. Interestingly, our data suggest incomplete viral replication, increased subgenomic RNA and fewer infectious viruses in paediatric cultures, indicating more defective viral genomes, presumably due to the strong interferon response. Similar observations have been made in animal challenge experiments^35^ and patient studies^36,37^, in which discrepancies between viral RNA and infectious viral load were also reported.

SARS-CoV-2 infection in older adult NECs led to epithelial damage and early signs of repair through cell migration and basal NEC proliferation. This was not observed in younger cultures. We also detected increases in ITGAV, ITGB6 and VIM proteins, which were attributed to the emergence of basaloid-like 2 cells. ITGB6 is expressed exclusively on epithelial cells but is virtually absent or expressed at very low levels in normal healthy adult epithelium^15^. It is highly upregulated in response to injury^38^ and is associated with fibrotic lung disease and epithelial cancers^17,39^.

Integrins also modulate cytokine expression and activate TGF-β1, implicated in fibrosis and EMT^38^, a process with distinct pathological roles in wound healing, tissue regeneration and organ fibrosis and cancer^40^. We hypothesize that age-dependent reprogramming of infected NECs contributes to COVID-19 pathogenesis by prolonging disease and enhancing viral spread.

SARS-CoV-2, influenza and other respiratory infections have previously been linked to dysregulated epithelial repair processes and disease pathogenesis^41–43^. We hypothesize that SARS-CoV-2 infection in older adult NECs leads to the emergence of the basaloid-like 2 cell type and drives EMT repair pathways. Elderly cultures infected with SARS-CoV-2 exhibit flattened epithelial tissue, decreased resistance and increased cell shedding, indicating EMT activation^44^. Such functional changes facilitate disease progression and potentially enhance viral spread^45,46^. Ultrastructural and immunofluorescence studies confirm significant SARS-CoV-2 infection in shed cells in severe COVID-19 cases^47^. Elevated vimentin levels, an EMT and basaloid cell marker, are also present in these cultures. Our immunofluorescent assays reveal unique vimentin cage-like structures known to recruit viral components for assembly and egress^26^. In addition, the virus may directly interact with ITGB6, a component of caveolae involved in viral internalization^38,48,49^. In vitro studies suggest that the SARS-CoV-2 spike protein interacts with integrins^50,51^, potentially serving as a viral entry route in non-ACE2-expressing cells, thereby promoting infection in older adults. These findings integrate into our proposed model, where older adult cultures are more prone to induce basaloid-like 2 cells in infection, and these cells support both viral spread and disease progression.

In summary, we have shown that SARS-CoV-2 shows age-specific tropism in nasal epithelial cells, targeting goblet cells in children and secretory cells in older adults. Paediatric cells exhibit a strong antiviral response, resulting in limited viral replication. Older adult cells undergo shedding and more epithelial damage. Altered repair pathways and an increase in basaloid-like 2 cells associated with fibrosis markers contribute to greater viral spread in older adults. These findings provide insights into age-related COVID-19 pathogenesis and demonstrate how impaired repair processes enhance SARS-CoV-2 infection in older individuals.

## Methods

### Participants and ethics

Participants were recruited from five large hospital sites in London, the United Kingdom: the Great Ormond Street Hospital NHS Foundation Trust, the University College London Hospitals NHS Foundation Trust, the Royal Free London NHS Foundation Trust (the Royal Free Hospital and the Barnet Hospital) and the Whittington Health NHS Trust from March 2020 to February 2021. All participants provided written informed consent. Ethics approval was given through the Living Airway Biobank, administered through the UCL Great Ormond Street Institute of Child Health (REC reference: 19/NW/0171, IRAS project ID: 261511, Northwest Liverpool East Research Ethics Committee). Exclusion criteria for the cohort included current smokers, active haematological malignancies or cancer, known immunodeficiencies, sepsis from any cause and blood transfusions within 4 weeks, known bronchial asthma, diabetes, hay fever and other known chronic respiratory diseases such as cystic fibrosis, interstitial lung disease and chronic obstructive pulmonary disease. Nasal brushings were obtained by trained clinicians from healthy paediatric (0–11 years), adult (30–50 years) and older adult (≥70 years) donors who tested negative for SARS-CoV-2 (within 24–48 h of sampling) and reported no respiratory symptoms in the preceding 7 weeks. Brushings were taken from the inferior nasal concha zone using cytological brushes (Scientific Laboratory Supplies, CYT1050). All methods were performed following the relevant guidelines and regulations. Details of the study population are shown in Supplementary Table 1.

### Differentiated human nasal epithelial cell culture

Human nasal brushings were collected fresh for this study and immediately placed in a 15 ml sterile Falcon tube containing 4 ml of transport medium (αMEM supplemented with 1× penicillin/streptomycin (Gibco, 15070), 10 ng ml^−1^ gentamicin (Gibco, 15710) and 250 ng ml^−1^ amphotericin B (ThermoFisher, 10746254)) on ice. Four matched paediatric nasal brush samples were sent directly for scRNA-seq^9^. To minimize sample variation, all samples were processed within 24 h of collection and cultured to P1 as previously described^52^. Briefly, biopsies were co-cultured with 3T3-J2 fibroblasts and Rho-associated protein kinase inhibitor (Y-27632) in epithelial cell expansion medium consisting of a 3:1 ratio DMEM:F12 (Gibco, 21765), 1× penicillin/streptomycin and 5% FBS (Gibco; 10270) supplemented with 5 μM Y-27632 (Cambridge Bioscience, Y1000), 25 ng ml^−1^ hydrocortisone (Sigma, H0888), 0.125 ng ml^−1^ EGF (Sino Biological, 10605), 5 μg ml^−1^ insulin (Sigma, I6634), 0.1 nM cholera toxin (Sigma, C8052), 250 ng ml^−1^ amphotericin B (Gibco, 10746254) and 10 μg ml^−1^ gentamicin (Gibco, 15710).

Basal cells were separated from the co-culture flasks by differential sensitivity to trypsin and seeded onto collagen I-coated, semi-permeable membrane supports (Transwell, 0.4 µm pore size, Corning). Cells were submerged for 24–48 h in an epithelial cell expansion medium, after which the apical medium was removed, and the basolateral medium was exchanged for epithelial cell differentiation medium to generate ‘air–liquid interface’ (ALI) conditions. PneumaCult ALI medium (STEMCELL Technologies, 05001) was used for differentiation media following manufacturer instructions. Basolateral media were exchanged in all cultures three times a week and maintained at 37 °C and 5% CO_2_. ALI cultures were maintained in PneumaCult ALI medium for 4 weeks to produce differentiated NECs for all downstream experimentation.

### Wound healing assay

Mechanical injury of NEC cultures was performed by aspiration in direct contact with the apical cell layer using a P200 sterile pipette tip, creating a wound with a diameter ranging from 750 to 1,500 μm. After wounding, the apical surface of the culture was washed with 200 μl PBS to remove cellular debris. The area of the wound was tracked with the aid of time-lapse microscopy with images taken every 60 min at ×4 magnification (Promon, AIS v.4.6.0.5.). The wound area was calculated each hour using ImageJ. The initial wound area was expressed as 100% to account for variability of wound size. Wounds were considered to be closed when the calculated area fell below 2%, the effective limit of detection due to image processing. Wound closure was calculated as follows: Wound closure (%) = 100 − ((Area/Initial Area) × 100). Wound closure (%) plotted as a function of time (h) was used to calculate the rate of wound closure (% h^−1^).

### Virus propagation

The SARS-CoV-2 isolate hCoV-19/England/2/2020 obtained from Public Health England (PHE) was used in this study. For virus propagation, the African green monkey kidney cell line Vero E6 (ATCC: CVCL_0574; a kind gift from The Francis Crick Institute, London, United Kingdom and authenticated for use in this study) was used. Vero E6 cells were maintained in DMEM supplemented with 5% FCS and 1× penicillin/streptomycin. Cell media were replenished three times a week and maintained at 37 °C and 5% CO2. Vero E6 cells were infected with an MOI of 0.01 p.f.u. per cell in serum-free DMEM supplemented with 1% NEAA, 0.3% (w/v) BSA and 1× penicillin/streptomycin. A mock condition was conducted in parallel in which an equivalent volume of PBS++ was used instead of viral inoculum. The viral and mock-inoculated cell media were collected after 48 h, centrifuged at 10,000 *g* for 10 min to remove cellular debris and stored at −70 °C. The viral titre was determined by plaque assay (see below).

### Viral infection of NEC cultures

After 28 days, NEC cultures were rinsed with sterile PBS++ and then infected with viral inoculum suspended in PBS++ (4.5 × 10^4^ p.f.u. ml^−1^, ~0.1 MOI) or an equivalent volume of mock inoculum suspended in PBS++ (mock infection) for 1 h on the apical compartment at 37 °C and 5% CO_2_. The virus inocula were then removed, and the NEC cultures were washed with sterile PBS++ and incubated for up to 72 h. This timepoint was chosen as maximum viral replication was observed at days 2–3 in our pilot studies (Extended Data Fig. 3a).

### Infectious viral load quantification by plaque assay

Vero E6 cells were grown to confluence on 24-well plates and then inoculated with serial dilutions of apical supernatant and cell lysates from infected cultures for 1 h at 37 °C and 5% CO_2_. The inoculum was replaced by an overlay medium supplemented with 1.2% (w/v) cellulose and incubated for 48 h at 37 °C and 5% CO_2_. Plates were fixed with 4% (w/v) paraformaldehyde for 30 min and overlay was aspirated from individual wells. Crystal violet staining was performed for a minimum of 20 min, and then plates were washed with water. The number of visible plaques was counted.

### Viral copy number quantification

Viral gene quantification was performed on apical wash supernatants from experiments. Samples were lysed in AVL buffer (Qiagen) and stored at −80 °C until further processing. Viral RNA extractions were performed using a QIAamp viral RNA kit (Qiagen) following manufacturer instructions. Extracted RNA samples (5 μl) were quantified in one-step RT–qPCR using AgPath-ID one-step RT–PCR (Applied Biosystems) with the following cycle conditions: 45 °C for 10 min, 95 °C for 15 min, (95 °C for 15 s + 58 °C for 30 s) in a total of 45 cycles.

Cellular gene quantification was performed with cultured cells collected at the end of the experiments. Cells were lysed in RLT buffer (Qiagen) and extraction was performed using an RNeasy mini kit (Qiagen) following manufacturer instructions. Total RNA was converted into cDNA with qScript cDNA supermix (Quantabio) following manufacturer instructions. RT–qPCR was performed using *Taq*Man Fast Advanced Master mix with the following cycle conditions: 50 °C for 2 min, 95 °C for 10 min, 95 °C for 30 s and 60 °C for 1 min in a total of 45 cycles. The expression was normalized with GAPDH and then presented as 2^−(ΔCт)^ in arbitrary units.

### SARS-CoV-2 genomic sequencing

#### Viral genome read coverage

To visualize the viral read coverage along the viral genome, we used the 10X Genomics cellranger barcoded binary alignment map (BAM) files for every sample. We filtered the BAM files to only retain reads mapping to the viral genome using the bedtools intersect tool^52^. We converted the BAM files into sequence alignment map (SAM) files to filter out cells that were removed in our single-cell data pre-processing pipeline. The sequencing depth for each base position was calculated using samtools count. To characterize read distribution along the viral genome, we counted transcripts of 10 different open reading frames (ORFs): ORF1ab, Surface glycoprotein (S), ORF3a, Envelope protein (O), Membrane glycoprotein (M), ORF6, ORF7a, ORF8, Nucleocapsid phosphoprotein (N) and ORF10.

#### Detection of SARS-CoV-2 subgenomic RNAs

Subgenomic RNA analysis was conducted using Periscope^53^. Briefly, Periscope distinguished sgRNA reads on the basis of the 5′ leader sequences being directly upstream from each gene’s transcription. The sgRNA counts were then normalized into a measure termed sgRPTL, by dividing the sgRNA reads by the mean depth of the gene of interest and multiplying by 1,000.

### Proteomics

#### Mass spectrometry

Paired mock- and SARS-CoV-2-infected airway surface fluids from groups of 10 paediatric, adult and older adult cultures were selected for this assay. For mass spectrometry, samples were inactivated with the KeyPro UV LED decontamination system (Phoseon Technology) before removal from the Biosafety level 3 laboratory (BSL3). Proteins were precipitated using ice-cold acetone. Protein pellets were resuspended in the digestion buffer as previously described and trypsin (Promega) digested to peptides^54^. Peptides were desalted by solid phase extraction (SPE) and separated by reverse phase chromatography on a NanoAquity LC system coupled to a SYNAPT G2-Si mass spectrometer (Waters) in a UDMSE positive ion electrospray ionization mode. Raw MS data were processed using Progenesis QI analysis software (Nonlinear Dynamics). Peptide identification was performed using the UniProt human reference proteome, with one missed cleavage and 1% peptide false discovery rate (FDR). Fixed modifications were set to carbamidomethylation of cysteines and dynamic modifications of oxidation of methionine.

#### Western blot

Samples were resolved on 4–15% Mini-PROTEAN TGX Precast Protein Gel (Bio-rad, 4561083) with high molecular mass standards of 10–250 kDa. Proteins were transferred to a Trans-Blot Turbo Mini 0.2 µm nitrocellulose membrane in a Trans-Blot Turbo Transfer System (Bio-rad, 1704150). Membranes were blocked in Odyssey blocking buffer overnight at 4 °C. Membranes were probed with primary antibodies described in Supplementary Table 2, with dilutions prepared in Odyssey blocking buffer. Incubation with primary antibodies was performed at room temperature (r.t.) for 1 h. These included rabbit anti-ACE2 recognizing both long and short isoforms (Abcam, ab15348, 1:2,000) and rabbit anti-ACE2 specific for the long isoform (Abcam, ab108252, 1:2,000), rat anti-alpha-tubulin (Sigma-Aldrich, MAB1864, 1:2,000) and acetylated forms (Sigma-Aldrich, T6793, 1:2,000), mouse anti-SARS-CoV-2 spike glycoprotein (Abcam, ab273433, 1:2,000), rabbit anti-GAPDH (Abcam, ab9485, 1:3,000), rabbit anti-vimentin (Abcam, ab16700, 1:500) and rabbit anti-E-cadherin (Abcam, ab40772, 1:10,000). After three 15 min washes in PBS containing 0.1% Tween 20, the membranes were incubated with the appropriate IRDye secondary antibodies: goat anti-mouse (LI-COR, 926-68070, dilution 1:18,000) and goat anti-rabbit (LI-COR, 926-32211, dilution 1:18,000), both at room temperature for 1 h. The blots were then visualized using an Odyssey CLx imager and quantified using Image Studio Lite software

#### Cytokine assay

Apical supernatants were collected by washing the apical surface with 200 μl of PBS. These were snap frozen at −70 °C and inactivated with the KeyPro UV LED decontamination system (Phoseon Technology) in the CL3 laboratory before handling them in a CL2 laboratory. Cytokine and chemokine levels were assessed in 25 μl of supernatants using the multiplex BD CBA bead-based immunoassay kits including: IL6: A7, 558276; IL8 (CXCL8): A9, 558277; TNFα: C4, 560112; IFNγ: E7, 558269; IP10 (CXCL10): B5, 558280; IFNα: B8, 560379; and IL10: B7, 558274. Data were acquired using the BD LSRII flow cytometer and concentrations were obtained from a standard curve (provided with the kit). Analysis was performed using the FCAP software (v.3.0, BD Biosciences).

### Microscopy

#### Immunofluorescence confocal microscopy

For immunofluorescence confocal imaging, NEC cultures were fixed using 4% (v/v) paraformaldehyde for 30 min, permeabilized with 0.2% Triton X-100 (Sigma) for 15 min and blocked using 5% goat serum (Sigma) in PBS for 1 h. The cultures were then incubated overnight at 4 °C with primary antibodies described in Supplementary Table 2, with dilutions prepared in 5% goat serum in PBS with 0.1% Triton X-100. The primary antibodies used included rabbit anti-ACE2 (Abcam, ab15348, diluted 1:200), mouse anti-MUC5AC (Sigma-Aldrich, MAB2011, diluted 1:500), rat anti-alpha-tubulin (tyrosinated) (Sigma-Aldrich, MAB1864, diluted 1:100), mouse anti-alpha-tubulin (acetylated) (Sigma-Aldrich, T6793, diluted 1:100), mouse anti-SARS-CoV-2 spike glycoprotein (Abcam, ab273433, diluted 1:500), rabbit anti-GAPDH (Abcam, ab9485, diluted 1:250), mouse anti-dsRNA (Jena Bioscience, RNT-SCI-10010500, diluted 1:100), rabbit anti-MX1 (Abcam, ab207414, diluted 1:250), rabbit anti-cytokeratin 5 conjugated with Alexa Fluor 647 (Abcam, ab193895, diluted 1:100), rabbit anti-vimentin (Abcam, ab16700, diluted 1:1,000), rabbit anti-IL28+29 (Abcam, ab191426, diluted 1:100), goat anti-BPIFA1 (Abcam, EB11482, diluted 1:100) and rat anti-integrin beta 6 (Abcam, ab97588, diluted 1:100).

Following primary antibody incubation, cultures were washed and then incubated with secondary antibodies diluted in 1.25% goat serum in PBS with 0.1% Triton X-100 at r.t. for 1 h the following day. The secondary antibodies included donkey anti-mouse Alexa Fluor 647 (Jackson ImmunoResearch, 715-605-151, 1:600), donkey anti-rat Alexa Fluor 647 (Jackson ImmunoResearch, 712-605-153, 1:600), donkey anti-mouse Alexa Fluor 594 (Jackson ImmunoResearch, 715-585-151, 1:600), donkey anti-rat Alexa Fluor 594 (Jackson ImmunoResearch, 712-585-153, 1:600), donkey anti-mouse Cy3 (Jackson ImmunoResearch, 715-165-151, 1:600), donkey anti-rabbit Cy3 (Jackson ImmunoResearch, 711-165-152, 1:600), donkey anti-rat Alexa Fluor 488 (Jackson ImmunoResearch, 712-545-153, 1:600), donkey anti-rabbit Alexa Fluor 488 (Jackson ImmunoResearch, 711-545-152, 1:600), donkey anti-mouse Alexa Fluor 488 (Jackson ImmunoResearch, 715-545-151, 1:600) and donkey anti-goat Alexa Fluor 488 (Jackson ImmunoResearch, 705-545-147, 1:600).

After the secondary antibody incubation, cultures were stained with Alexa Fluor 555 phalloidin (ThermoFisher, A34055, 4 μg ml^−1^) for 1 h and DAPI (Sigma, 2 μg ml^−1^) for 15 min at r.t. to visualize F-actin and nuclei, respectively. Samples were washed three times with PBS containing 0.1% Tween 20 after each incubation step.

Imaging was carried out using an LSM710 Zeiss confocal microscope, and the resulting images were analysed using Fiji/ImageJ v.2.1.0/153c54 for metrics including mean intensity, cell protrusion, culture thickness, % signal coverage, wound area and pseudocolouring^55^. Intensity profiles were generated using Nikon NIS-Elements analysis module, and three-dimensional (3D) renderings of immunofluorescence images were produced with Imaris software (Bitplane, Oxford Instruments; v.9.5/9.6).

#### Transmission electron microscopy

Cultured NECs that were either SARS-CoV-2-infected or non-infected were fixed with 4% paraformaldehyde and 2.5% glutaraldehyde in 0.05 M sodium cacodylate buffer at pH 7.4 and placed at 4 °C for at least 24 h. The samples were incubated in 1% aqueous osmium tetroxide for 1 h at r.t. before subsequent en bloc staining in undiluted UA-Zero (Agar Scientific) for 30 min at r.t. The samples were dehydrated using increasing concentrations of ethanol (50, 70, 90, 100%), followed by propylene oxide and a mixture of propylene oxide and araldite resin (1:1). The samples were embedded in araldite and left at 60 °C for 48 h. Ultrathin sections were acquired using a Reichert Ultracut E ultramicrotome and stained using Reynold’s lead citrate for 10 min at r.t. Images were taken on a JEOL 1400Plus TEM equipped with an Advanced Microscopy Technologies (AMT) XR16 charge-coupled device (CCD) camera and using the AMT Capture Engine software.

### Sample preparation for single-cell RNA sequencing

Cultured NECs were processed using an adapted cold-active protease single-cell dissociation protocol^56^, as described below, based on a previously used protocol^9^ to allow for a better comparison of matched samples included in both studies. In total, NECs derived from *n* = 3 paediatric, 4 adult and 4 older adult donors were processed at 24, 48 and 72 h post infection (SARS-CoV-2 and mock) for scRNA-seq.

First, the transwell inserts, in which the NEC cultures were grown, were carefully transferred into a new 50 ml Falcon tube and any residual transport medium was carefully removed so as not to disturb the cell layer. Dissociation buffer (2 ml) was then added to each well, ensuring the cells were covered; 10 mg ml^−1^ protease from *Bacillus licheniformis* (Sigma-Aldrich, P5380) and 0.5 mM EDTA in HypoThermosol (STEMCELL Technologies, 07935). The cells were incubated on ice for 1 h. Every 5 min, cells were gently triturated using a sterile blunt needle, decreasing from a 21G to a 23G needle. Following dissociation, protease was inactivated by adding 400 µl of inactivation buffer (HBSS containing 2% BSA) and the cell suspension was transferred to a new 15 ml Falcon tube. The suspension was centrifuged at 400 *g* for 5 min at 4 °C and the supernatant was discarded. Cells were resuspended in 1 ml dithiothreitol wash (10 mM dithiothreitol in PBS) (ThermoFisher, R0861) and gently mixed until any remaining visible mucous appears to break down, or for ~2–4 min. The mixture was centrifuged at 400 *g* for 5 min at 4 °C and the supernatant was removed. The cells were resuspended in 1 ml of wash buffer (HBSS containing 1% BSA) and centrifuged once more under the same conditions. The single-cell suspension was then filtered through a 40 µm Flowmi cell strainer. Finally, the cells were centrifuged and resuspended in 30 µl of resuspension buffer (HBSS containing 0.05% BSA). Using trypan blue, total cell counts and viability were assessed. Cells (3,125) were then pooled together from the 4 biological replicates with corresponding conditions (for example, all mock viral treatments at 24 h) and the cell concentration was adjusted for 7,000 targeted cell recovery according to the 10x Chromium manual (between 700–1,000 cells per µl). The pools were then processed immediately for 10 × 5′ single-cell capture using the Chromium Next GEM Single Cell V(D)J reagent kit v.1.1 (Rev E Guide) or the Chromium Next GEM Single Cell 5′ V2 (Dual index) kit (Rev A guide). Each pool was run twice.

Of note, each sample was processed fresh for 5’ Next Gen single-cell RNA sequencing and thus pooled per timepoint when loading on the 10X chromium controller. Extra steps were taken where possible to balance sex, age as well as technical factors (that is, 10X chromium kit versions) within these sample pools. Furthermore, the downstream process of the sample pools, including library preparation and sequencing (see below) contained samples from the 4 h, 24 h and 72 h timepoints to mitigate additional technical effects. Timepoints can be seen to be well mixed within the single-cell dataset as visualized via a uniform manifold approximation and projection (UMAP) in Extended Data Fig. 1b.

For several samples (Supplementary Table 3), 1 µl viral RT oligo (either at 5 µM or 100 µM, PAGE) was spiked into the master mix (at step 1.2.b in the 10X guide, giving a final volume of 75 µl) to help with the detection of SARS-CoV-2 viral reads. The samples were then processed according to manufacturer instructions, with the viral cDNA separated from the gene expression libraries (GEX) by size selection during step 3.2. Here the supernatant was collected (159 µl) and transferred to a new PCR tube and incubated with 70 µl of SPRI beads (0.6× selection) at r.t. for 5 min. The SPRI beads were then washed according to the guide and the viral cDNA was eluted using 30 µl of EB buffer. Neither changes to the transcriptome were previously observed upon testing the addition of viral oligo^9^, nor were any significant changes observed with an increasing concentration upon comparison, outside of a small increase in the overall number of SARS-CoV-2 reads detected. The RT oligo sequence was as follows: 5′-AAGCAGTGGTATCAACGCAGAGTACTTACTCGTGTCCTGTCAACG-3′

### Library generation and sequencing

The Chromium Next GEM Single Cell 5′ V2 kit (v.2.0 chemistry) was used for single-cell RNA-seq library construction. For all NEC culture samples, libraries were prepared according to manufacturer protocol (10X Genomics) using individual Chromium i7 sample indices. GEX libraries were pooled and sequenced on a NovaSeq 6000 S4 flow cell (paired-end, 150 bp reads), aiming for a minimum of 50,000 paired-end reads per cell for GEX libraries.

### Single-cell RNA-seq data processing

#### Computational pipelines, processing and analysis

The single-cell data were mapped to a GRCh38 ENSEMBL 93 derived reference, concatenated with 21 viral genomes (featuring SARS-CoV-2), of which the NCBI reference sequence IDs are: NC_007605.1 (EBV1), NC_009334.1 (EBV2), AF156963 (ERVWE1), AY101582 (ERVWE1), AY101583 (ERVWE1), AY101584 (ERVWE1), AY101585 (ERVWE1), AF072498 (HERV-W), AF127228 (HERV-W), AF127229 (HERV-W), AF331500 (HERV-W), NC_001664.4 (HHV-6A), NC_000898.1 (HHV-6B), NC_001806.2 (herpes simplex virus 1), NC_001798.2 (herpes simplex virus 2), NC_001498.1 (measles morbillivirus), NC_002200.1 (mumps rubulavirus), NC_001545.2 (rubella), NC_001348.1 (varicella zoster virus), NC_006273.2 (cytomegalovirus) and NC_045512.2 (SARS-CoV-2). When examining viral load per cell type, we first removed ambient RNA by SoupX^57^. The alignment, quantification and preliminary cell calling of NEC culture samples were performed using the STARsolo functionality of STAR v.2.7.3a, with the cell calling subsequently refined using the Cell Ranger v.3.0.2 version of EmptyDrops^58^. Initial doublets were called on a per-sample basis by computing Scrublet scores^59^ for each cell, propagating them through an over-clustered manifold by replacing individual scores with per-cluster medians and identifying statistically significant values from the resulting distribution, replicating previous approaches^60,61^.

#### Quality control, normalization and clustering

Mixed genotype samples were demultiplexed using Souporcell^62^ and reference genotypes. DNA from samples was extracted following manufacturer protocol (Qiagen, DNeasy blood and tissue kit 69504 and Qiagen Genomic DNA miniprep kit) and single nucleotide polymorphism (SNP) array-derived genotypes generated by Affymetrix UK Biobank Axiom Array kit by Cambridge Genomic Services (CGS). Cells that were identified as heterotypic doublets by Souporcell were discarded. Quality control was performed on SoupX-cleaned expression matrixes. Genes with fewer than 3 counts and cells with more than 30% mitochondrial reads were filtered out. Cells with a scrublet score >0.3 and adjusted *P* value < 0.8 were predicted as doublets and filtered out. Expression values were then normalized to a sum of 1 × 10^4^ per cell and log-transformed with an added pseudocount of 1. Highly variable genes were selected using the scanpy.pp.highly_variable_genes() function in Scanpy. Principal component analysis (PCA) was performed and the top 30 principal components were selected as input for l. We performed graph-based batch integration with the bbknn method^63^ using experimental pools and chemistry as batch covariate (encoded as ‘bbknn_batch’ in the object). Clustering was performed with the Leiden^64^ algorithm on a *k*-nearest-neighbour graph of a PCA space derived from a log(counts per million/100 + 1) representation of highly variable genes, according to the Scanpy protocol^65^. Leiden clustering with a resolution of 1 was used to separate broad cell types (basal, goblet, secretory). For each broad cell type, clustering was then repeated, starting from highly variable gene discovery to achieve a higher resolution and a more accurate separation of refined cell types. Annotation was first performed automatically using a Celltypist^66^ model built on the in vivo dataset of nasal airway brushes^9^, and then using manual inspection of each of the clusters and further manual annotation using known airway epithelial marker genes.

### Developmental trajectory inference

Pseudotime inference was performed on the whole object or the basal/goblet compartment using Monocle 3 (refs. ^67,68^). Briefly, a cycling basal cell was chosen as a ‘root’ cell for the basal compartment, showing the highest combined expression of *KRT5*, *MKI67* and *NUSAP1* genes. For the goblet compartment, a goblet 1 cell was chosen as a root, showing the highest combined expression of *TFF3*, *SERPINB3*, *MUC5AC*, *MUC5B* and *AQP5*. Cells were grouped into different clusters using the group_cells() function, learning the principal graph using the learnGraph() function and ordering cells along the trajectory using the ordercells() function. A second pseudotime was inferred with Palantir (1.0.1)^69^. The cycling basal ‘root’ cell was determined as above and an unsupervised pseudotime inference was performed on a Scanpy-derived diffusion map. The five inferred endpoints were inspected and three were deemed to be very closely biologically related and replaced with a joint endpoint with the highest combined expression of *OMG*, *PIFO* and *FOXJ1*. The pseudotime inference was repeated with the two remaining inferred endpoints and the marker derived one serving as the three terminal states.

### Differential abundance analysis

To determine cell states that are enriched in the SARS-CoV-2 versus mock conditions for the different age groups, we used the Milo framework for differential abundance analysis using cell neighbourhoods^14^. Briefly, we computed *k*-nearest-neighbour graphs of cells in the whole dataset using the buildGraph() function, assigned cells to neighbourhoods using the makeNhoods() function and counted the number of cells belonging to each sample using the countCells() function. Each neighbourhood was assigned the original cluster labels using majority voting. To test for enrichment of cells in the SARS-CoV-2 condition versus the mock condition, we modelled the cell count in neighbourhoods as a negative binomial generalized linear model, using a log-linear model to model the effects of age and treatment on cell counts (logFC). We control for multiple testing using the weighted Benjamini–Hochberg correction as described in ref. ^14^ (spatialFDR correction). Neighbourhoods were considered enriched in SARS-CoV-2 vs mock if the spatialFDR < 0.1 and logFC > 0.

### Expression signature analysis

To determine the enrichment of basaloid or interferon genes in the annotated clusters, we used the Scanpy function scanpy.tl.score_genes() to score the gene signature for each cell. The gene list for computing the basaloid score was composed of the *EPCAM, CDH1, VIM, FN1, COL1A1, CDH2, TNC, VCAN, PCP4, CUX2, SPINK1, PRSS2, CPA6, CTSE, MMP7, MDK, GDF15, PTGS2, SLCO2A1, EPHB2, ITGB8, ITGAV, ITGB6, TGFB1, KCNN4, KCNQ5, KCNS3, CDKN1A, CDKN2A, CDKN2B, CCND1, CCND2, MDM2, HMGA2, PTCHD4* and *OCIAD2* genes. The gene list for computing the IFN alpha score was composed of the *ADAR, AXL, BST2, EIF2AK2, GAS6, GATA3, IFIT2, IFIT3, IFITM1, IFITM2, IFITM3, IFNAR1, IFNAR2, KLHL20, LAMP3, MX2, PDE12, PYHIN1, RO60, STAR* and *TPR* genes and the gene list for computing the IFN gamma score was composed of the *OAS3, OASL, OTOP1, PARP14, PARP9, PDE12, PIAS1, PML, PPARG, PRKCD, PTAFR, PTPN2, RAB20, RAB43, RAB7B, RPL13A, RPS6KB1, SHFL, SIRPA, SLC11A1, SLC26A6, SLC30A8, SNCA, SOCS1, SOCS3, SP100, STAR, STAT1, STX4, STX8, STXBP1, STXBP3, STXBP4, SUMO1, SYNCRIP, TDGF1, TLR2, TLR3, TLR4, TP53, TRIM21, TRIM22, TRIM25, TRIM26, TRIM31, TRIM34, TRIM38, TRIM5, TRIM62, TRIM68, TRIM8, TXK, UBD, VAMP3, VCAM1, VIM, VPS26B, WAS, WNT5A, XCL1, XCL2, ZYX* genes, as used in ref. ^9^.

### Gene set enrichment analysis

Wilcoxon rank-sum test was performed to determine differentially expressed genes between clusters using the scanpy.tl.rank_genes_groups() function. Differentially expressed genes were further analysed using GSEA via ShinyGO^70^.

### In vivo sub-analysis

Sex- and age-matched healthy adults and paediatric airway samples (*n* = 10 total) were subsetted from our previous dataset^9^ for label transfer of the in vitro cell annotation using CellTypist as described above. Selected sample IDs from the in vivo dataset are shown in Supplementary Table 4. These were selected to match the mean age and range, and sex of the current study as the sample collection and processing were conducted in parallel between studies.

### In vivo integration

We performed integration of 8 single-cell datasets comprising 614,695 cells from upper and lower airways from healthy and COVID-19 patients from paediatric (0–18 years), adult (19–50 years) and older adult (51–90 years) samples^9,10,12,18–22^. Expression values were then normalized to a sum of 1 × 10^4^ per cell and log-transformed with an added pseudocount of 1. Highly variable genes were selected using the Scanpy function scanpy.pp.highly_variable_genes(). PCA was performed and the top 30 principal components were selected as input for Harmony^71^ to correct for batch effects between studies and compute a batch-corrected *k*-nearest-neighbour graph. The clustering was performed with the Leiden^64^ algorithm on a *k*-nearest-neighbour graph of a PCA space derived from a log(counts per million/100 + 1) representation of highly variable genes, according to the Scanpy protocol^65^. Leiden clustering with a resolution of 1 was used to separate broad cell types (basal, goblet, secretory) and subclustering was used for more accurate separation of fine-grained cell types. Annotation was first performed automatically using a Celltypist^66^ model built on the in vivo dataset of nasal airway brushes^9^, and then using manual inspection of each of the clusters and further manual annotation using known epithelial marker genes.

### Statistical analysis on in vivo dataset

Due to the large proportions of zero counts, we fitted zero-inflated Poisson (ZIP) models to the counts of nasal epithelial cells including the natural logarithm of the total number of cells per donor as an offset in the models for both basaloid and gobletInFam cells. This allowed us to estimate both the incidence of nasal epithelial cells () and the probability of a donor being in the zero counts class () as functions of disease and age groups. We first included interaction terms between the disease and age groups in both the incidence and the zero counts parts of the models. Generalized additive models for location, scale and shape were fitted using the packages gamlss^72^ and glmmTMB^73^ (to include random effects on the probability of zero class by donor), both in the R language and environment for statistical computing (v.4.2.3)^74^. Using the Bayesian Information Criterion (BIC) as a goodness-of-fit statistic, we decided to include the main effects of disease in both the linear predictors for incidence and probability of belonging to the zero class after stratifying by age group.

### Statistical analysis

Statistical analysis was performed using R or GraphPad Prism 9 and details of statistical tests used are indicated. Data distribution was assumed to be normal unless stated differently, and a Kruskal–Wallis test was used to test for homogeneity of locations using R. The determination of sample sizes was guided by those established in previous scRNA-seq and studies using ALI cultures^9,75^, rather than through the application of specific statistical methods. In total, NEC cultures generated from 11 participants were used to create our in vitro single-cell dataset, including 3 paediatric (<12 years), 4 adult (30–50 years) and 4 older adult (>70 years) donors. Additional statistical power here was provided by the experimental design, sampling from multiple timepoints (4, 24 and 72 h post SARS-CoV-2 infection), with the inclusion of matched mock-infected controls for each. Samples were also carefully pooled (see Methods) to help avoid batch effects and run across multiple lanes on the 10X controller. Together, this resulted in a total of 66 NEC samples processed for single-cell sequencing, with a total of 139,598 high-quality cells sequenced. Further validation of our in vitro single-cell data and our key observation was provided using an integrated in vivo single-cell dataset (using published patient datasets) and numerous experimental validation assays. Data collection and analysis were not performed blind to the conditions of the experiments. Representative images are displayed as examples for quantified data (1*n* of the total *n* noted in their corresponding summary graphs) unless otherwise stated in the figure legend.

### Reporting summary

Further information on research design is available in the Nature Portfolio Reporting Summary linked to this article.

### Supplementary information

Supplementary Information Supplementary Figs. 1–3.

Reporting Summary

Supplementary Tables Supplementary Tables 1–4.

### Source data

Source Data Figs. 1–6 Statistical source data.

Source Data Extended Data Figs. 1–8 and 10 Statistical source data.

Source Data Extended Data Fig. 9 Statistical source data and unprocessed Western blots.

## Data Availability

RNA-seq data are available in the European Genome-Phenome Archive (EGA) (https://ega-archive.org/) under accession number EGAD00001015345. The datasets from our study can be downloaded and explored interactively through a web portal (https://covid19cellatlas.org, https://www.covid19cellatlas.org/ALI_COVID19/in-vitro/, https://www.covid19cellatlas.org/ALI_COVID19/in-vivo/). Quality control metrics for our single-cell data are provided on the web portal page. All other data needed to evaluate the conclusions are available within the Article or its Supplementary Information. Viral sequences resulting from this study can be found on Genbank under the accession numbers PP346398–PP346416. Source image files for the main figures are available via Figshare at 10.6084/m9.figshare.25193618.v1 (ref. ^76^). Source image files for the extended figures are available via Figshare at 10.6084/m9.figshare.25194005.v1 (ref. ^77^). Source image files for supplementary data are available via Figshare at 10.6084/m9.figshare.25196396.v1 (ref. ^78^). Source data are provided with this paper.

## Code availability

Custom code for the analysis performed in this study is publicly available via GitHub at https://github.com/Teichlab/ALI_COVID19 (ref. ^79^). Free data access to the single-cell objects used in this study is available at https://www.covid19cellatlas.org/.
