# Author Correction: Age-specific nasal epithelial responses to SARS-CoV-2 infection

**DOI:** 10.1038/s41564-024-01757-z

**Published:** 2024-06-18

**Authors:** Maximillian N. J. Woodall, Ana-Maria Cujba, Kaylee B. Worlock, Katie-Marie Case, Tereza Masonou, Masahiro Yoshida, Krzysztof Polanski, Ni Huang, Rik G. H. Lindeboom, Lira Mamanova, Liam Bolt, Laura Richardson, Batuhan Cakir, Samuel Ellis, Machaela Palor, Thomas Burgoyne, Andreia Pinto, Dale Moulding, Timothy D. McHugh, Aarash Saleh, Eliz Kilich, Puja Mehta, Chris O’Callaghan, Jie Zhou, Wendy Barclay, Paolo De Coppi, Colin R. Butler, Mario Cortina-Borja, Heloise Vinette, Sunando Roy, Judith Breuer, Rachel C. Chambers, Wendy E. Heywood, Kevin Mills, Robert E. Hynds, Sarah A. Teichmann, Kerstin B. Meyer, Marko Z. Nikolić, Claire M. Smith

**Affiliations:** 1 Great Ormond Street UCL Institute of Child Health, London, UK; 2 Wellcome Sanger Institute, Cambridge, UK; 3 UCL Respiratory, Division of Medicine, University College London, London, UK; 4 UCL Institute of Ophthalmology, University College London, London, UK; 5 Royal Brompton Hospital, Guy’s and St Thomas’ NHS Foundation Trust, London, UK; 6 UCL Centre for Clinical Microbiology, Division of Infection and Immunity, University College London, London, UK; 7 Royal Free Hospital NHS Foundation Trust, London, UK; 8 University College London Hospitals NHS Foundation Trust, London, UK; 9 Department of Infectious Disease, Imperial College London, London, UK; 10 Great Ormond Street Hospital NHS Foundation Trust, London, UK; 11 Epithelial Cell Biology in ENT Research (EpiCENTR) Group, Developmental Biology and Cancer Department, Great Ormond Street UCL Institute of Child Health, University College London, London, UK; 12 UCL Cancer Institute, University College London, London, UK; 13 Theory of Condensed Matter, Cavendish Laboratory/Dept Physics, University of Cambridge, Cambridge, UK

**Keywords:** SARS-CoV-2, Molecular biology, Mechanisms of disease

Correction to: *Nature Microbiology* 10.1038/s41564-024-01658-1, published online 15 April 2024

In the version of the article initially published, Paolo De Coppi’s surname originally appeared as “DeCoppi”. In the third paragraph of the article, the callout to Extended Data Fig. 3i has been amended from Extended Data Fig. 4i. In the legend to Fig. 6, the description of panel m (“Summary figure highlighting the key findings from the study. Created with BioRender.com”) was missing and has now been added. In the “Statistical analysis” paragraph in the Methods, the text now reading “a Kruskal–Wallis test was used to test for homogeneity of locations using R” originally read “… normality using R”. These corrections have been made to the HTML and PDF versions of the article.

