# Supplementary material for: Age-specific nasal epithelial responses to SARS-CoV-2 infection
Source: Nat Microbiol. 2024 Apr 15;9(5):1293–311. doi: 10.1038/s41564-024-01658-1 (PMC11087271; doi:10.1038/s41564-024-01658-1)
Supplement: Supplementary file 1 — Supplementary Figs. 1–3. [file 41564_2024_1658_MOESM1_ESM.pdf]

nature microbiology

Article https://doi.org/10.1038/s41564-024-01658-1

Age-specific nasal epithelial responses to SARS-CoV-2 infection

In the format provided by the authors and unedited

Supplementary Figure 1

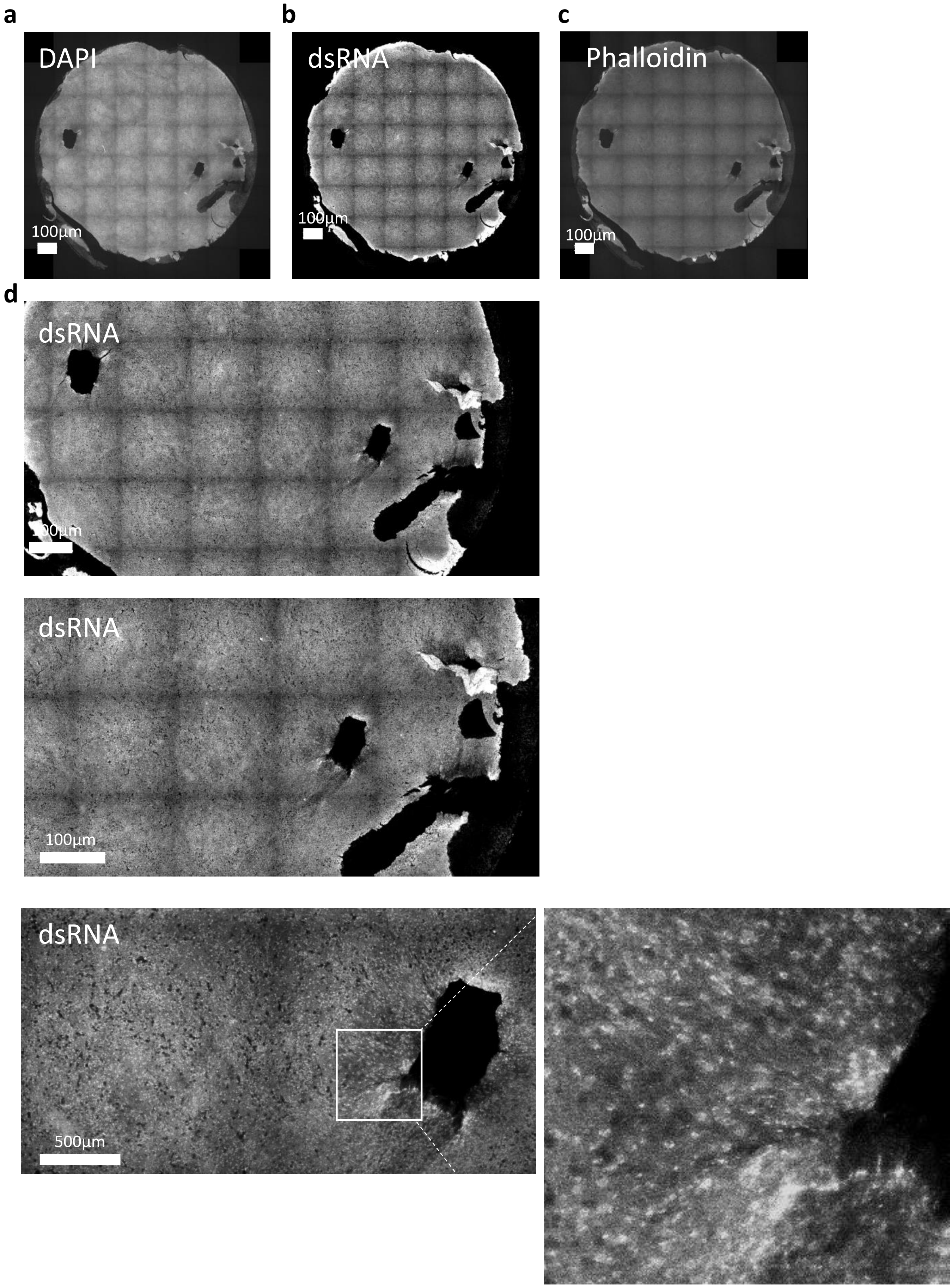

Supplementary Figure 1. Whole well scan of NEC culture. Immunofluorescent image scan of a whole mounted older adult NEC culture 24h p.i and wounded, stained with **(a)** DAPI, **(b)** anti-dsRNA and **(c)** phalloidin. **(d)** Zoomed in images of NEC culture, showing increase dsRNA staining around wound edge site. Scale differs and scale bars are given on each image in white.

Supplementary Figure 2

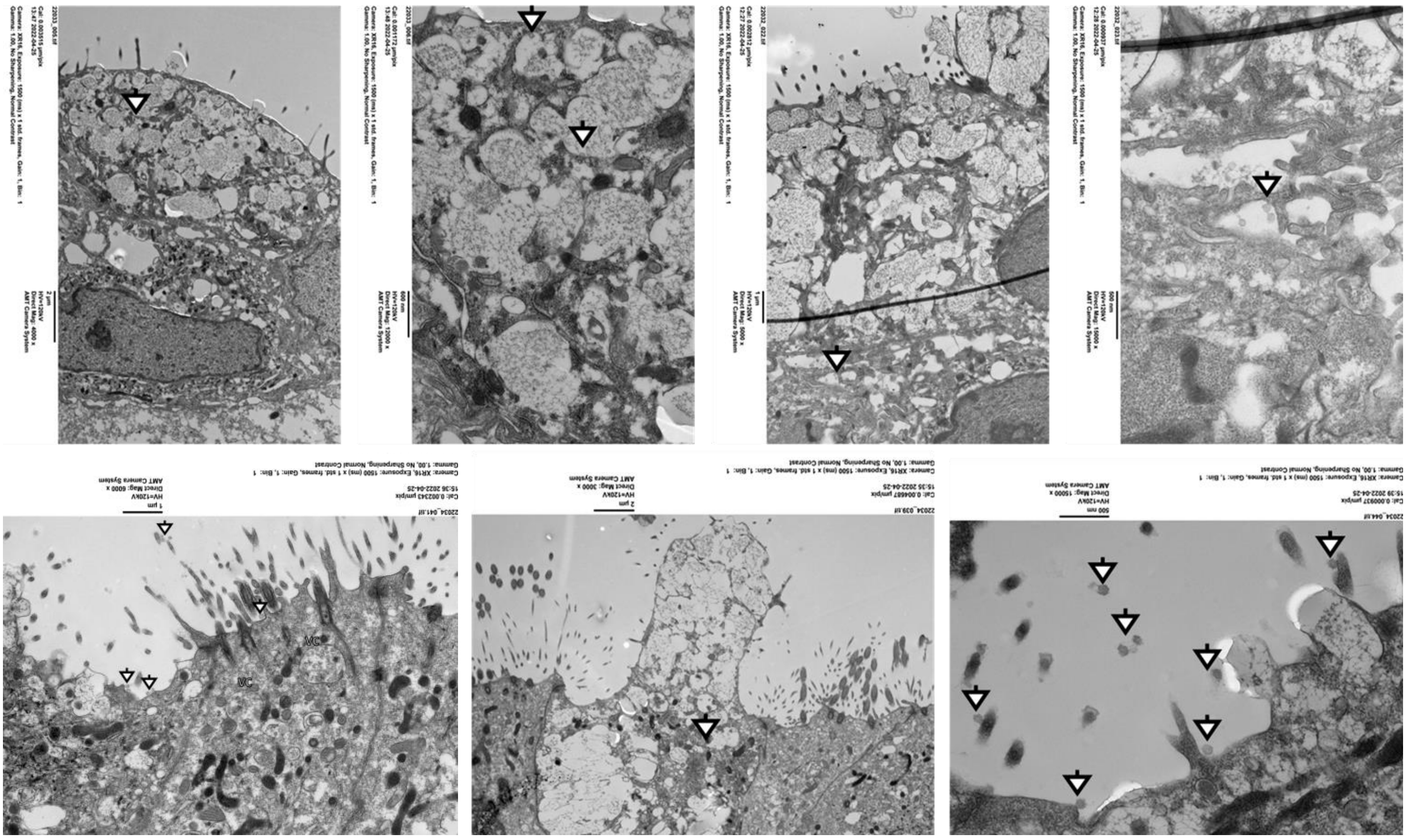

Supplementary Figure 2. Transmission electron micrographs of Paediatric Goblet 2 inflammatory cells. Transmission electron micrographs of Goblet cells from a paediatric donor 72h p.i. with SARS-CoV-2. The panels show different magnifications, the scale bar is given on the left (top panels) or above (bottom panels) of each image. Viral particles are indicated with white arrows.

Supplementary Figure 3

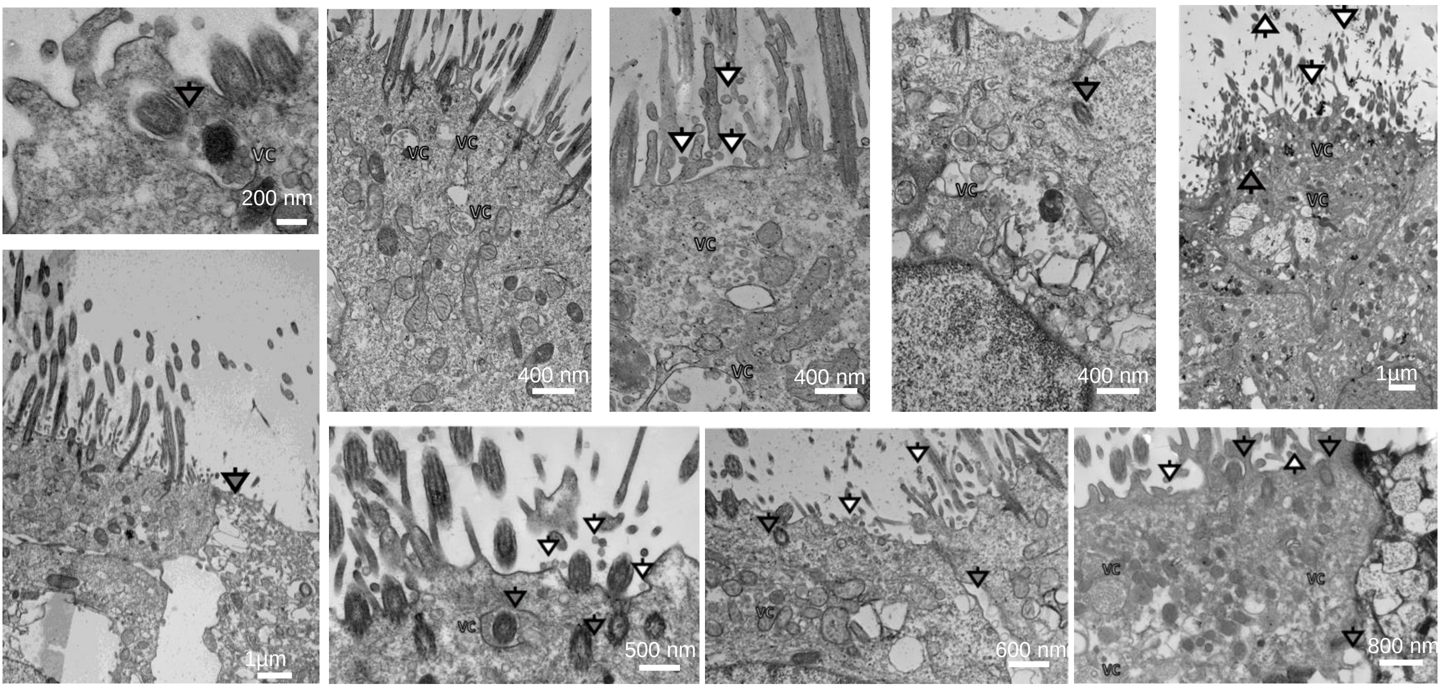

Supplementary Figure 3. Transmission electron micrograph of epithelial cell damage. Transmission electron micrograph of epithelial cell damage at 72h p.i with SARS-CoV-2: Virions (white arrows), viral compartments (VC), Cytopathology including endocytosed cilia basal bodies, loss of tight junctions (grey arrows). Scale differs and scale bars are given on each image in white.
