# Supplementary material for: Age-specific nasal epithelial responses to SARS-CoV-2 infection
Source: Nat Microbiol. 2024 Apr 15;9(5):1293–311. doi: 10.1038/s41564-024-01658-1 (PMC11087271; doi:10.1038/s41564-024-01658-1)
Supplement: Supplementary file 2 — Reporting Summary [file 41564_2024_1658_MOESM2_ESM.pdf]

nature portfolio

Corresponding author(s): Sarah Teichmann, Kerstin Meyer, Marko Z. Nikolic and Claire M Smith

Last updated by author(s): Feb 9, 2024

Reporting Summary

Nature Portfolio wishes to improve the reproducibility of the work that we publish. This form provides structure for consistency and transparency in reporting. For further information on Nature Portfolio policies, see our Editorial Policies and the Editorial Policy Checklist.

Statistics

For all statistical analyses, confirm that the following items are present in the figure legend, table legend, main text, or Methods section.

n/a	Confirmed	
☐	☒	The exact sample size (*n*) for each experimental group/condition, given as a discrete number and unit of measurement
☐	☒	A statement on whether measurements were taken from distinct samples or whether the same sample was measured repeatedly
☐	☒	The statistical test(s) used AND whether they are one- or two-sided<br>*Only common tests should be described solely by name; describe more complex techniques in the Methods section.*
☐	☒	A description of all covariates tested
☐	☒	A description of any assumptions or corrections, such as tests of normality and adjustment for multiple comparisons
☐	☒	A full description of the statistical parameters including central tendency (e.g. means) or other basic estimates (e.g. regression coefficient) AND variation (e.g. standard deviation) or associated estimates of uncertainty (e.g. confidence intervals)
☐	☒	For null hypothesis testing, the test statistic (e.g. *F*, *t*, *r*) with confidence intervals, effect sizes, degrees of freedom and *P* value noted<br>*Give P values as exact values whenever suitable.*
☐	☒	For Bayesian analysis, information on the choice of priors and Markov chain Monte Carlo settings
☐	☒	For hierarchical and complex designs, identification of the appropriate level for tests and full reporting of outcomes
☐	☒	Estimates of effect sizes (e.g. Cohen's *d*, Pearson's *r*), indicating how they were calculated

Our web collection on statistics for biologists contains articles on many of the points above.

Software and code

Policy information about availability of computer code

Data collection	No specific code was used in the data collection
Data analysis	*Provide a description of all commercial, open source and custom code used to analyse the data in this study, specifying the version used OR state that no software was used.*

For manuscripts utilizing custom algorithms or software that are central to the research but not yet described in published literature, software must be made available to editors and reviewers. We strongly encourage code deposition in a community repository (e.g. GitHub). See the Nature Portfolio guidelines for submitting code & software for further information.

Data

Policy information about availability of data

All manuscripts must include a data availability statement. This statement should provide the following information, where applicable:

- Accession codes, unique identifiers, or web links for publicly available datasets
- A description of any restrictions on data availability
- For clinical datasets or third party data, please ensure that the statement adheres to our policy

Provide your data availability statement here.

Human research participants

Policy information about studies involving human research participants and Sex and Gender in Research.

Reporting on sex and gender	We ensured all samples were age and sex matched.
Population characteristics	Population characteristics are described in Table 1
Recruitment	Participants were recruited from five large hospital sites in London, UK, namely Great Ormond Street Hospital NHS Foundation Trust, University College London Hospitals NHS Foundation Trust, Royal Free London NHS Foundation Trust (Royal Free Hospital and Barnet Hospital) and Whittington Health NHS Trust from March 2020 to February 2021. Exclusion criteria for the cohort included current smokers, active haematological malignancies or cancer, known immunodeficiencies, sepsis from any cause and blood transfusions within 4 weeks, known bronchial asthma, diabetes, hay fever, and other known chronic respiratory diseases such as cystic fibrosis, interstitial lung disease and chronic obstructive pulmonary disease. Nasal brushings were obtained by trained clinicians from healthy paediatric (0–11 years), adult (30-50 years) and elderly (≥70 years) donors that tested negative for SARS-CoV-2 (within 24-48 hours of sampling) and reported no respiratory symptoms in the preceding 7 weeks. All methods were performed in accordance with the relevant guidelines and regulations.
Ethics oversight	Ethical approval was given through the Living Airway Biobank, administered through the UCL Great Ormond Street Institute of Child Health (REC reference: 19/NW/0171, IRAS project ID: 261511, Northwest Liverpool East Research Ethics Committee).

Note that full information on the approval of the study protocol must also be provided in the manuscript.

Field-specific reporting

Please select the one below that is the best fit for your research. If you are not sure, read the appropriate sections before making your selection.

☒ Life sciences ☐ Behavioural & social sciences ☐ Ecological, evolutionary & environmental sciences

For a reference copy of the document with all sections, see nature.com/documents/nr-reporting-summary-flat.pdf

Life sciences study design

All studies must disclose on these points even when the disclosure is negative.

Sample size	Samples sizes were calculated based on our previous studies using ALI cultures grown from donor material from different disease groups. Using a conservative estimated minimum effect size of 40% for a difference in viral titres between age groups (children defined as <11, adults aged between 30-50 and elderly >70) with an estimated standard deviation of 30%, a desired power of 80% and a p-value of < 0.05 for statistical significance, a sample size is eight donors per group per experiment was needed.<br><br>For scRNAseq samples sizes have been calculated based on our previous work to analyse epithelial tissue from COVID19 patients. This work determined that a sample size of twelve donors per age group per experimental condition (mock-infected, SARS-CoV2 infected) is adequate.
Data exclusions	UCL8 was of insufficient quality and was excluded from the scRNAseq analysis
Replication	*Describe the measures taken to verify the reproducibility of the experimental findings. If all attempts at replication were successful, confirm this OR if there are any findings that were not replicated or cannot be reproduced, note this and describe why.*
Randomization	As this was not a clinical trial, randomisation was not relevant for this study
Blinding	As this was not a clinical trial, blinding was not relevant and the statistical tests were performed on paired samples from same individual

Reporting for specific materials, systems and methods

We require information from authors about some types of materials, experimental systems and methods used in many studies. Here, indicate whether each material, system or method listed is relevant to your study. If you are not sure if a list item applies to your research, read the appropriate section before selecting a response.

Materials & experimental systems

n/a	Involved in the study
	☒ Antibodies
	☒ Eukaryotic cell lines
☒	Palaeontology and archaeology
☒	Animals and other organisms
☒	Clinical data
☒	Dual use research of concern

Methods

n/a	Involved in the study
☒	ChIP-seq
☒	Flow cytometry
☒	MRI-based neuroimaging

Antibodies

Antibodies used	Anti-ACE2 (L+S) Rabbit IgG (ab15328) ; Anti-Mucin MUC5AC Mouse IgG (MAB2011) ;Anti-Tubulin Rat IgG2a (MAB1864) ; Anti-Tubulin Mouse IgG2b (T6793) ; Anti-SARS Spike Mouse IgG1 (ab273433) ; dsRNA Mouse IgG2a (jenabioscience.com RNT-SCI-10010200) ; Cytokeratin 5 (conjugation:AlexaF647) Rabbit IgG (ab193895) ; DNAI2 Mouse IgG2a (H00064446-M01) ; GAPDH Rabbit IgG (ab9485) ; E-Cadherin Rabbit (ab40772) ; Vimentin Rabbit (ab16700) ; N-Cadherin Mouse (ab40772) ; Rat anti-integrin alpha v+beta 6 Abcam
Validation	All antibodies used were commercial antibodies.

Eukaryotic cell lines

Policy information about cell lines and Sex and Gender in Research

Cell line source(s)	Murine embryonic 3T3-J2 fibroblasts originally sourced from Simon Broad, Prof. Fiona Watt (Kings College London, London, UK), Dr. Paola Bonfanti (University College London, London, UK), and Prof. Howard Green (Harvard Medical School, Boston, MA)
Authentication	*Describe the authentication procedures for each cell line used OR declare that none of the cell lines used were authenticated.*
Mycoplasma contamination	All cell lines and primary cells were confirmed negative for mycoplasma contamination by PCR testing
Commonly misidentified lines (See ICLAC register)	none
