# Supplementary material for: Age-specific nasal epithelial responses to SARS-CoV-2 infection
Source: Nat Microbiol. 2024 Apr 15;9(5):1293–311. doi: 10.1038/s41564-024-01658-1 (PMC11087271; doi:10.1038/s41564-024-01658-1)
Supplement: Supplementary file 3 — Supplementary Tables 1–4. [file 41564_2024_1658_MOESM3_ESM.docx]

**Supplementary Tables**

**Supplementary Table 1.  Study population**

| **Study Population​** | | **​** | **Total cultures ​**  **obtained (n)​** | **Total cells for**  **scRNAseq​** |
| --- | --- | --- | --- | --- |
| Total n​ | 29​ | ​ | 251​ | ​ |
| % Female​ | 41%​ | ​ | 38%​ | ​ |
| **Brushings​** | **n​** | **Age (mean ±SD)​** | **n​** | ​ |
| Paediatric (0-11y)​ | 14​ | 4.9 ±4.2​​ | 118​ | 35,892 |
| Adult (30-50y)​ | 9​ | 36.9 ±2.7​​ | 65​ | ​56,221 |
| Older Adults  (70y+)​ | 9​ | 83.6 ±6.7​​ | 68​ | ​50,485 |
|  |  |  | **Total cells** | **139,598** |

**Supplementary Table 2. Antibodies used in this stidy**

Primaries for ICC

| **Antigen/Protein target​** | **Host​** | **Supplier​** | **Product Code ​** | **Dilution (ICC)​**  **​** | **Dilution (WB)​** |
| --- | --- | --- | --- | --- | --- |
| ACE2 (Long+Short)​ | Rabbit​ | Abcam​ | ab15348​ | 1:200​ | 1:2000​ |
| ACE2 (Long)​ | Rabbit​ | Abcam​ | ab108252​ | N/A​ | 1:2000​ |
| MUC5AC​ | Mouse​ | Sigma-Aldrich (Merck)​ | MAB2011​ | 1:500​ | N/A​ |
| Alpha-Tubulin (tyrosinated)​ | Rat​ | Sigma-Aldrich (Merck)​ | MAB1864​ | 1:100​ | 1:2000​ |
| Alpha-Tubulin (acetylated)​  ​ | Mouse​ | Sigma-Aldrich (Merck)​ | T6793​ | 1:100​ | 1:2000​ |
| SARS-CoV-2 Spike Glycoprotein​ | Mouse​ | Abcam​ | ab273433​ | 1:500​ | 1:2000​ |
| GAPDH​ | Rabbit​ | Abcam​ | ab9485​ | 1:250​ | 1:3000​ |
| dsRNA antibody ​ | Mouse​ | Jena Bioscience​ | RNT-SCI-10010500​ | 1:100​ | N/A​ |
| MX1​ | Rabbit​ | Abcam​ | ab207414​ | 1:250​ | N/A​ |
| Cytokeratin 5 (conjugation:AlexaF647)​ | Rabbit​ | Abcam​ | ab193895​ | 1:100​ | N/A​ |
| E-Cadherin​ | Rabbit​ | Abcam​ | ab40772​ | N/A​ | 1:10000​ |
| Vimentin​ | Rabbit​ | Abcam​ | ab16700​ | 1:1000​ | 1:500​ |
| IL28+29​ | Rabbit​ | Abcam​ | ab191426​ | 1:100​ | N/A​ |
| BPIFA1​ | Goat​ | Abcam​ | EB11482​ | 1:100​ | N/A​ |
| Integrin beta 6 (avB6)​ | Rat ​ | Abcam​ | ab97588​ | 1:100​ | N/A​ |

Secondaries for ICC

| **Conjugate​** | **Target​** | **Host​** | **Clonality​** | **Ex/Em​** | **Product Code​** | **Dilution​** |
| --- | --- | --- | --- | --- | --- | --- |
| Alexa Fluor 647​ | Mouse​ | Donkey​ | Polyclonal​ | 651-667nm​ | 715-605-151​ | 1:600​ |
| Alexa Fluor 647​ | Rat​ | Donkey​  ​ | Polyclonal​  ​ | 651-667nm​  ​ | 712-605-153​ | 1:600​  ​ |
| Alexa-Fluor 594​ | Mouse​ | Donkey​ | Polyclonal​  ​ | 591-614nm​ | 715-585-151​ | 1:600​  ​ |
| Alexa-Fluor 594​ | Rat​ | Donkey​ | Polyclonal​ | 591-614nm​ | 712-585-153​ | 1:600​  ​ |
| Cy3​ | Mouse​ | Donkey​ | Polyclonal​ | 550-550nm*​ | 715-165-151​ | 1:600​  ​ |
| Cy3​ | Rabbit​ | Donkey​ | Polyclonal​ | 550-550nm*​ | 711-165-152​ | 1:600​  ​ |
| Alexa Fluor 488​ | Rat​ | Donkey​ | Polyclonal​ | 493-519nm​ | 712-545-153​ | 1:600​  ​ |
| Alexa Fluor 488​ | Rabbit​ | Donkey​ | Polyclonal​ | 493-519nm​ | 711-545-152​ | 1:600​  ​ |
| Alexa Fluor 488​ | Mouse​ | Donkey​ | Polyclonal​ | 493-519nm​ | 715-545-151​ | 1:600​  ​ |
| Alexa Fluor 488​ | Goat​ | Donkey​ | Polyclonal​ | 493-519nm​  ​ | 705-545-147​ | 1:600​  ​ |

Secondaries for Western

| **Conjugate​** | **Target​** | **Host​** | **Clonality​** | **Ex/Em​** | **Product code​** | **Dilution​** |
| --- | --- | --- | --- | --- | --- | --- |
| IRDye 680RD​ | Mouse​ | Goat​ | Polyclonal​ | 676-694nm​ | 926-68070​ | 1:18,000​ |
| IRDye800CW​ | Rabbit​ | Goat​ | Polyclonal​ | 778-795nm​ | 926-32211​ | 1:18,000​  ​ |

**Supplementary Table 3.  Samples processed for scRNAseq**

| **Lab sample ID** | **Samples in pool** | **10x Kit** | **Viral Oligo spiked** |
| --- | --- | --- | --- |
| ALI1_mock_4h | 276 mock 4h | v1.1 and v2 | NA |
|  | 291 mock 4h |  |  |
|  | 354 mock 4h |  |  |
|  | 357 mock 4h |  |  |
| ALI1_SARS_4h | 276 SARS 4h | v1.1 and v2 | NA |
|  | 291 SARS 4h |  |  |
|  | 354 SARs 4h |  |  |
|  | 357 SARS 4h |  |  |
| ALI1_mock_24h | 276 mock 24h | v1.1 and v2 | NA |
|  | 291 mock 24h |  |  |
|  | 354 mock 24h |  |  |
|  | 357 mock 24h |  |  |
| ALI1_SARS_4h | 276 SARS 24h | v1.1 and v2 | NA |
|  | 291 SARS 24h |  |  |
|  | 354 SARS 24h |  |  |
|  | 357 SARS 24h |  |  |
| ALI1_mock_72h | 276 mock 72h | v1.1 and v2 | NA |
|  | 291 mock 72h |  |  |
|  | 354 mock 72h |  |  |
|  | 357 mock 72h |  |  |
| ALI1_SARS_72h | 276 SARS 72h | v1.1 and v2 | NA |
|  | 291 SARS 72h |  |  |
|  | 354 SARS 72h |  |  |
|  | 357 SARS 72h |  |  |
| ALI2_mock4h | 901 mock 4h | v1.1 | 1uL @ 5μM |
|  | 902 mock 4h |  |  |
|  | 905 mock 4h |  |  |
|  | 907 mock 4h |  |  |
| ALI2_SARS4h | 901 SARS 4h | v1.1 | 1uL @ 5μM |
|  | 902 SARS 4h |  |  |
|  | 905 SARs 4h |  |  |
|  | 907 SARS 4h |  |  |
| ALI3_mock4h | 903 mock 4h | v1.1 | 1uL @ 5μM |
|  | 906 mock 4h |  |  |
|  | 926 mock 4h |  |  |
|  | 927 mock 4h |  |  |
| ALI3_SARS4h | 903 SARS 4h | v1.1 | 1uL @ 5μM |
|  | 906 SARS 4h |  |  |
|  | 926 SARS 4h |  |  |
|  | 927 SARS 4h |  |  |
| ALI2_mock24h | 901 mock 24h | v1.1 | 1uL @ 5μM |
|  | 902 mock 24h |  |  |
|  | 905 mock 24h |  |  |
|  | 907 mock 24h |  |  |
| ALI2_SARS24h | 901 SARS 24h | v1.1 | 1uL @ 5μM |
|  | 902 SARS 24h |  |  |
|  | 905 SARS 24h |  |  |
|  | 907 SARS 24h |  |  |
| ALI3_mock24h | 903 mock 24h | v1.1 | 1uL @ 5μM |
|  | 906 mock 24h |  |  |
|  | 926 mock 24h |  |  |
|  | 927 mock 24h |  |  |
| ALI3_SARS24h | 903 SARS 24h | v1.1 | 1uL @ 5μM |
|  | 906 SARS 24h |  |  |
|  | 926 SARS 24h |  |  |
|  | 927 SARS 24h |  |  |
| ALI2_mock72h | 901 mock 72h | v1.1 | 1uL @ 5μM |
|  | 902 mock 72h |  |  |
|  | 905 mock 72h |  |  |
|  | 907 mock 72h |  |  |
| ALI2_SARS72h | 901 SARS 72h | v1.1 | 1uL @ 5μM |
|  | 902 SARS 72h |  |  |
|  | 905 SARs 72h |  |  |
|  | 907 SARS 72h |  |  |
| ALI3_mock72h | 903 mock 72h | v1.1 | 1uL @ 5μM |
|  | 906 mock 72h |  |  |
|  | 926 mock 72h |  |  |
|  | 927 mock 72h |  |  |
| ALI3_SARS72h | 903 SARS 72h | v1.1 | 1uL @ 5μM |
|  | 906 SARS 72h |  |  |
|  | 926 SARS 72h |  |  |
|  | 927 SARS 72h |  |  |
| ALI3_SARS4h_V2 | 903 SARS 4h | v2 | 1uL @ 100μM |
|  | 906 SARS 4h |  |  |
|  | 926 SARS 4h |  |  |
|  | 927 SARS 4h |  |  |
| ALI3_SARS24h_V2 | 903 SARS 24h | v2 | 1uL @ 100μM |
|  | 906 SARS 24h |  |  |
|  | 926 SARS 24h |  |  |
|  | 927 SARS 24h |  |  |
| ALI3_SARS72h_V2 | 903 SARS 72h | v2 | 1uL @ 100μM |
|  | 906 SARS 72h |  |  |
|  | 926 SARS 72h |  |  |
|  | 927 SARS 72h |  |  |

**Supplementary Table 4.  Donor demographics of samples used in the *in vivo* sub-analysis**

| **Donor ref​** | **Age​** | **Sex​** | **Age Group​** | **Number of cells​** | **Past medical history​** |
| --- | --- | --- | --- | --- | --- |
| **NP-10​** | 5y 11month​ | M​ | Paediatric​ | 2882​ | Cardiomyopathy​ |
| **NP-14​** | 6 weeks​ | F​ | Paediatric​ | 1816​ | Supraglottic cyst​ |
| **NP-16​** | 3y 11month​ | F​ | Paediatric​ | 5158​ | Non-verbal​ |
| **NP-22​** | 3 month​ | F​ | Paediatric​ | 405​ | Laryngeal cleft​ |
| **NP-31​** | 6y 9 month​ | M​ | Paediatric​ | 3046​ | unknown​ |
| **AN-9​** | 36y​ | F​ | Adult​ | 1359​ | non-smoker​ |
| **AN-11​** | 38y​ | F​ | Adult​ | 5309​ | ex-smoker (3 years)​ |
| **AN-12​** | 67y​ | F​ | Adult​ | 348​ | non-smoker​ |
| **AN-13​** | 55y​ | M​ | Adult​ | 4738​ | non-smoker​ |
| **AN-14​** | 52y​ | M​ | Adult​ | 837​ | non-smoker​ |
|  |  |  |  |  |  |
