# Supplementary material for: Age-specific nasal epithelial responses to SARS-CoV-2 infection
Source: Nat Microbiol. 2024 Apr 15;9(5):1293–311. doi: 10.1038/s41564-024-01658-1 (PMC11087271; doi:10.1038/s41564-024-01658-1)

Extended data 9b Western Blot

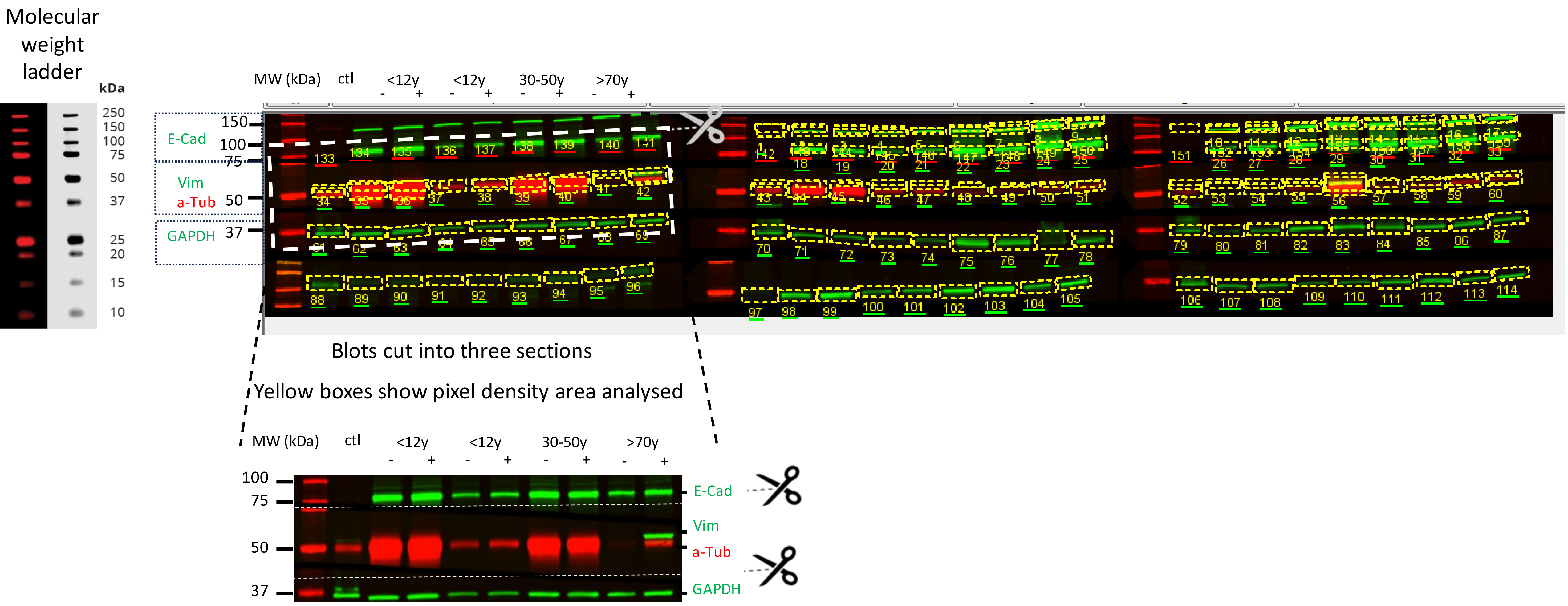

Supplement: Supplementary file 6 — Statistical source data and unprocessed Western blots. [file 41564_2024_1658_MOESM6_ESM.pdf]
